# Enhancing the thermostability of lignin peroxidase: Heme as a keystone cofactor driving stability changes in heme enzymes

**DOI:** 10.1016/j.heliyon.2024.e37235

**Published:** 2024-08-30

**Authors:** Joo Yeong Park, Seunghyun Han, Doa Kim, Trang Vu Thien Nguyen, Youhyun Nam, Suk Min Kim, Rakwoo Chang, Yong Hwan Kim

**Affiliations:** a School of Energy and Chemical Engineering, Ulsan National Institute of Science and Technology (UNIST), 50, UNIST-gil, Ulsan, 44919, Republic of Korea; b Department of Applied Chemistry, University of Seoul, 163, Seoulsiripdae-ro, Seoul, 02504, Republic of Korea; c Graduate School of Carbon Neutrality, Ulsan National Institute of Science and Technology (UNIST), 50, UNIST-gil, Ulsan, 44919, Republic of Korea

**Keywords:** Lignin peroxidase, Protein thermal stability, Enzymatic lignin conversion

## Abstract

Heme-containing enzymes, critical across life's domains and promising for industrial use, face stability challenges. Despite the demand for robust industrial biocatalysts, the mechanisms underlying the thermal stability of heme enzymes remain poorly understood. Addressing this, our research utilizes a ‘keystone cofactor heme-interaction approach’ to enhance ligand binding and improve the stability of lignin peroxidase (LiP). We engineered mutants of the white-rot fungus *Pc*LiP (*Phanerochaete chrysosporium*) to increase thermal stability by 8.66 °C and extend half-life by 29 times without losing catalytic efficiency at 60 °C, where typically, wild-type enzymes degrade. Molecular dynamics simulations reveal that an interlocked cofactor moiety contributes to enhanced structural stability in LiP variants. Additionally, a stability index developed from these simulations accurately predicts stabilizing mutations in other *Pc*LiP isozymes. Using milled wood lignin, these mutants achieved triple the conversion yields at 40 °C compared to the wild type, offering insights for more sustainable white biotechnology through improved enzyme stability.

## Highlights

• *Pc*LiP01 mutants demonstrate increased thermostability for biomass conversion.
• *Pc*LiP01 mutants show superior thermal properties over WT.
• The molecular dynamics simulation model precisely predicts *Pc*LiP isozyme stability.
• Mutants enhance milled wood lignin conversion to DMBQ at 40 °C.
• Research emphasizes heme cofactors in biocatalyst stability for lignin valorization.

## Graphical abstract

**Figure:**
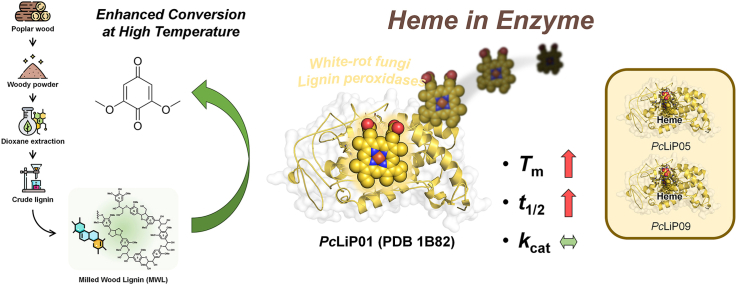

## 1 Introduction

White biotechnology focuses on sustainable chemical production using biocatalysts and biomass, such as lignin, to address the climate crisis [1]. Biocatalytic reactions in this field are promising due to their selectivity and energy efficiency under mild conditions. However, many enzymes, optimized for natural environments, lose their activity or stability under harsh industrial conditions, posing significant challenges for their industrial deployment. Recognizing the importance of enzyme modification for industrial applications, significant research has focused on enhancing the stability, activity, and substrate specificity of enzymes [2]. Particularly, improvements in thermal stability directly impact enzyme applications in biomass conversion, reducing the costs associated with enzyme usage and lowering the operational expenses in the biotechnology sector.

Lignin, a major component of plant biomass, is an underutilized aromatic polymer due to its complex structure. Efficiently converting lignin into valuable bioproducts under mild conditions is a pivotal challenge [3,4]. Heme-containing proteins, with their strong oxidizing abilities, are promising candidates for such biochemical transformations. They play a crucial role in sustaining life across almost all organisms, related to their essential functions in oxygen transport, electron transfer, and versatile oxidizing abilities [5]. Despite their wide range of applications, heme proteins share common challenges related to their optimum reaction conditions—typically around pH 5.0 to 6.0 and temperatures between 25 °C and 50°C—and their stability against substrates like H_2_O_2_ [6,7].

Various approaches were being explored to enhance the thermal stability of enzymes, including creating disulfide bonds, salt bridges or glycosylation [[8], [9], [10]]. For heme enzymes specifically, enhancements through modifications such as those applied to horseradish peroxidase and lignin peroxidase (LiP) are critical for their effective use in lignin biomass conversion [[11], [12], [13], [14]].

This study aims to fill this gap by focusing on LiP from *Phanerochaete chrysosporium*, a heme-containing enzyme noted for its high potential in lignin degradation and applications in flavor enhancement, food quality, cosmetics, environmental cleanup, and bioenergy [15,16]. Enhancing LiP's thermal stability is critical for its deployment in the industrial conversion of lignin biomass. Our research leverages a novel approach, examining the role of the heme cofactor in stabilizing the overall structure of LiP, akin to a keystone in arch architecture. By conducting a phylogenetic tree analysis based on structural similarities among heme enzymes and classifying them into groups based on their melting temperatures (*T*_m_), we aim to uncover key residues that contribute to thermal stability.

Employing mutation studies and molecular dynamics (MD) simulations, we investigate the mechanisms by which these key residues, in conjunction with the heme cofactor, influence LiP's thermal stability. Based on this, we demonstrated that the mutants with enhanced thermal stability decompose lignin biomass more efficiently than the wild-type. This comprehensive approach not only enhances our understanding of heme protein stability but also demonstrates the practical applications of these enzymes in the efficient conversion of biomass, contributing significantly to the field of white biotechnology.

## 2 Materials and methods

### 2.1 Materials

*Escherichia coli* (*E. coli*) BL21(DE3) (Real Biotech Corporation, Taiwan) and the pET-21b(+) expression vector (Novagen, USA) were used for protein overexpression. Isopropyl β-D-1-thiogalactopyranoside (IPTG), Luria-Bertani (LB) broth high salt, and ampicillin sodium for the cultivation and induction of gene expression were purchased from Duchefa Biochemie, Netherlands. Trizma base, Trizma hydrochloride, sodium hydroxide, urea, L-glutathione oxidized, hemin, calcium chloride (CaCl_2_), potassium chloride, boric acid, veratryl alcohol (VA) and hydrogen peroxide (H_2_O_2_) purchased from Sigma‒Aldrich were used without further purification. Acetic acid was obtained from Junsei, Japan. Guanidine hydrochloride, sodium acetate, and phosphoric acid were purchased from Tokyo Chemical Industry, Japan. VivaFlow 200 flipflow filtration (10,000 MWCO Hydrosart) was purchased from Sartorius for the concentration of enzyme solution. The HiTrap Q HP anion exchange column used for protein purification was procured from GE Healthcare BioSciences, USA. Protein Thermal Shift dye kit was purchased from Applied Biosystems, USA.

### 2.2 Expression and purification of recombinant PcLiP in E. coli host

Site-directed mutagenesis (SDM) was conducted for the construction of *Pc*LiP mutants. Polymerase chain reactions (PCRs) were performed in 50 μl volumes containing 200 ng of template DNA, 50 pg of each forward and reverse primer, 2.5 units of *Pfu* DNA polymerase (Bioneer), and 1 × FailSafe PreMix G (Lucigen). The sequences of primers are listed in Table S1. The PCR protocol included an initial denaturation of 5 min at 95 °C, followed by 15 cycles of three steps: 1 min at 95 °C, 1 min at a temperature of 5 °C lower than the melting temperature of primers, and 7 min at 68 °C, concluding with a final extension of 30 min at 68 °C. The amplified products were purified using a PCR purification kit (GeneAll), the template DNA was eliminated via *Dpn*I digestion, and the product was transformed into *E. coli* DH5α (Real Biotech Corporation) cells without further purification. The introduction of the mutations was verified through sequencing using T7 promoter and terminator primers.

For the main culture, a 200 ml medium composed of 10 g l^−1^ NaCl, 20 g l^−1^ tryptone, and 10 g l^−1^ yeast extract was prepared in a 1 l flask. In other instances, LB high salt was used as the medium. Overall procedures for enzyme preparation are the same as the previous publication [17]. Briefly, insolubly expressed *Pc*LiPs in the inclusion body form were collected after cell lysis. The inclusion body was dissolved in 8 M urea and refolded overnight in the refolding buffer containing hemin. The refolded protein solution was concentrated with a tangential flow filtration system and dialyzed with sodium acetate buffer of pH 4 and pH 6. Enzyme was purified with HiTrap Q HP anion column and ÄKTA Pure fast protein liquid chromatography system, and fractions showing the highest Reinheitszahl (RZ) values (A_409 nm_/A_280 nm_), especially over 2.5, were collected. Variants with an RZ value not exceeding 2.5 despite repeated purification attempts, were excluded from further analysis due to insufficient heme content and low data reliability. The concentration of purified enzymes was determined by measuring the absorbance of the heme Soret band and its extinction coefficient (*ε*_409 nm_ = 168 mM^−1^ cm^−1^) [18] or by bicinchoninic acid assay in the case of apoprotein.

### 2.3 Determination of thermodynamic stability of PcLiP wild type and mutants by differential scanning fluorimetry (DSF)

The *T*_m_ of the enzymes was determined using a Protein Thermal Shift dye kit in real-time PCR system (Thermo Fisher Scientific, USA). The samples were prepared as per the manufacturer's protocol. In a 20 μl reaction mixture, 2 μg protein, 2.5 μl Protein Thermal Shift buffer and dye was added to 0.1 M BR buffer at pH 3.0. To ensure a clear fluorescence signal increase for each *Pc*LiP isozyme, the dye ratio was adjusted to higher concentrations than recommended in the manual (5-fold for *Pc*LiP01 and 9.375-fold for *Pc*LiP05 and *Pc*LiP09). The mixtures were centrifuged briefly and then heated from 25 to 99 °C at a rate of 0.05 °C s^−1^ in the real-time PCR system. Melt curve analysis were performed using Protein Thermal Shift Software v1.4. Each measurement was replicated four times.

### 2.4 Activity assay of PcLiP

The specific activity of *Pc*LiP was assessed by monitoring absorbance at 310 nm using a UV-1900i spectrophotometer (Shimadzu). The reaction mixture consisted of 0.02 μM enzyme and 2 mM VA as substrate, in a 0.1 M Britton-Robinson (BR) buffer at pH 3.0 and 25 °C. The reaction was initiated by adding 250 μM H_2_O_2_ and monitored over 30 s. The production of veratraldehyde was quantified using its extinction coefficient (*ε*_310 nm_ = 9.3 mM^−1^ cm^−1^) [19]. One unit of *Pc*LiP activity (U, μmol min^−1^) was defined as the amount of enzyme that catalyzes the conversion of 1 μmol VA to veratraldehyde per minute. Each measurement was replicated three times.

### 2.5 Determination of catalytic properties of PcLiP wild type and mutants

The catalytic properties were determined at 25 °C and 60 °C with varying concentrations of VA, ranging from 50 to 2000 μM. The enzyme and H_2_O_2_ concentrations were maintained at 0.02 μM and 250 μM, respectively. Specific activity was measured in 0.1 M BR buffer (pH 3.0) with each VA concentration. Kinetic parameters were estimated using SigmaPlot software.

### 2.6 Determination of thermal stability of PcLiP wild type and mutants by measuring residual activity after high-temperature incubation

*Pc*LiPs, diluted to 2 μM, were incubated at 55 °C in a water bath. At designated time points, their incubation was halted by transferring the tubes to a 25 °C water bath. Residual activities were measured as described above. The deactivation constant (*k*_d_) was estimated by a linear regression of ln(residual activity) against time (*t*), and the half-life (*t*_1/2_) was calculated using the equation *t*_1/2_ = (ln 2)/*k*_d_.

### 2.7 Milled wood lignin (MWL) depolymerization by PcLiP01

Poplar milled wood lignin (MWL) was sourced from Professor Joon Weon Choi's team at Seoul National University, prepared by the Bjorkman method [20]. Briefly, air-dried poplar cell walls were milled at 4 °C, extracted with 95 % dioxane, and stirred at room temperature. After centrifugation, the solution was evaporated, re-dissolved, precipitated, and freeze-dried. The final yield was about 20 %, based on the original lignin content. The enzymatic degradation of MWL was performed as follows: 1.5 mg ml^−1^ MWL, 2 μM *Pc*LiP01 WT, V181A or E40S/V181A, with intermittently supplied H_2_O_2_ (50 μM for every 1 h) in 10 ml BR buffer (100 mM, pH 3) at 40 °C. The concentration of 2,6-dimethoxy-1,4-benzoquinone (DMBQ) produced in the reaction was determined by HPLC (Agilent) using an Eclipse XBD-C18 column (4.6 × 150 mm, 3.5 μm, Agilent) at time points 0, 1, 2, 4, 6, 8 and 10 h.

### 2.8 Molecular dynamics (MD) simulation of PcLiP wild type and mutants

Molecular dynamics (MD) simulations of *Pc*LiPs were conducted using the CHARMM36m force field at room temperature (25 °C) and 80 °C with graphic processing unit (GPU)-accelerated OpenMM software [21,22]. The coordinates of *Pc*LiP01 WT were derived from the crystal structure (PDB ID: 1B82), and structures of other isozymes were generated by AlphaFold2 [23] and protonated at pH 3.0 using PDB2PQR [24], as described in detail in the next section. Mutants of *Pc*LiPs were constructed on an *in-silico* basis by substituting the corresponding residues with different amino acids using CHARMM-GUI [[25], [26], [27]]. The protonation states of the amino acids at pH 3.0 were calculated with PropKa 3.1 [28,29]. Each system was initially solvated in an 89 × 89 × 89 Å^3^ simulation box. Initial system configurations, including physiological conditions (0.15 M KCl), terminal and hydrogen patches, heme coordination, and periodic boundary conditions, were set up using CHARMM-GUI [[25], [26], [27]].

Each system underwent an initial energy minimization for 5000 steps, followed by equilibration for 10 ps in the NVT ensemble (*T* = 0 K) and subsequently for 340 ps in the NPT ensemble (*P* = 1 bar), gradually heating to 25 °C or 80 °C. The Langevin dynamics and isotropic Monte Carlo methods were employed as the thermostat and the barostat, respectively. During heating, the harmonic spring restraint (*k* = 400 kJ mol^−1^ nm^−2^) was applied to the alpha carbon (Cα) of each residue and gradually reduced to prevent protein denaturation. The stability of each system was monitored using root-mean-square deviation (RMSD) of proteins excluding N- and C-terminal residues (−7 to −1 and 329 to 344). Subsequently, MD simulations for each system were conducted for at least 20 ns without any restraints to Cα atoms. From the simulation results, early trajectories with unstable RMSD profiles were considered equilibration steps and were discarded, and the following trajectories showing stable RMSD trends were used for further analysis.

### 2.9 Generation of protein structures of PcLiP isozymes

The three-dimensional (3D) structures of *Pc*LiP isozymes were generated using AlphaFold2 [23]. The prediction parameters remained as the default settings of AlphaFold2 v2.1.0, with the exception of the number of central processing units (CPUs) being increased to 48. The AlphaFold2 source code can be accessed at https://github.com/deepmind/alphafold. The protonation states of each amino acid, excluding the heme iron-coordinating histidine, in the structures with the highest prediction scores were modified using PDB2PQR [24] at pH 3.0, employing the AMBER force field and naming scheme. A heme molecule and calcium ions were integrated into these structures by superimposing them with the crystal structure of *Pc*LiP01 using Discovery Studio software (BIOVIA). The final structures were subsequently utilized for generating MD coordinates via CHARMM-GUI.

### 2.10 Determination of heme-amino acid non-bond interactions from MD simulations

**Scheme 1:**
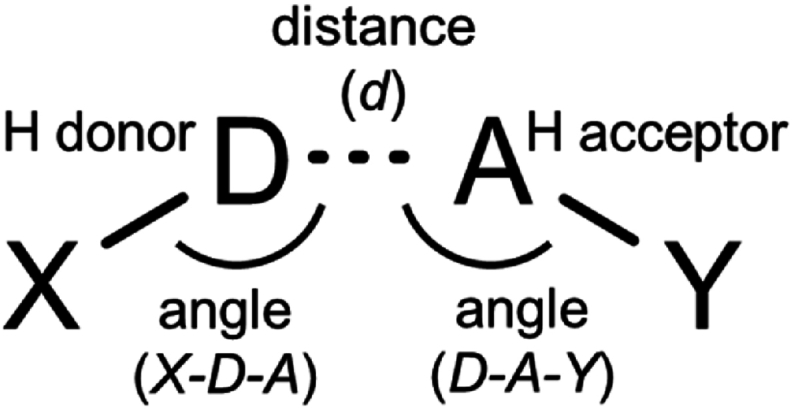
Angles and distance to determine non-bond interactions. The hydrogen atom to mediate non-bond interactions is not displayed.

After MD simulations, the trajectory data (DCD files) were analyzed to assess non-bond interactions between heme propionate groups and amino acid residues. Utilizing Discovery Studio (BIOVIA) software guidelines and relevant literature, criteria were set for identifying hydrogen bonds [30,31], carbon hydrogen bonds [32], and salt bridges [31]. Scheme 1 provides a visual overview of these interactions. Key parameters included the distance (*d*) and angles (*X-D-A* and *D-A-Y*) for potential (carbon) hydrogen bonds and salt bridges involving amino acid residues and heme propionate groups. The potential hydrogen donors of the amino acids backbone are the alpha carbon (Cα) atoms, and donor candidates of the amino acids sidechain are detailed in Table S2. Interactions were considered as (carbon) hydrogen bonds if *d* ≤ 3.4 Å for nitrogen or oxygen donors (or ≤ 3.8 Å for sulfur or carbon donors), and angles ranged from 90 to 180°. Additionally, interactions involving arginine or lysine were classified as salt bridges if *d* ≤ 5.6 Å. The frequency and distance of these interactions, categorized by amino acid sidechains and backbones, were quantified for further analysis.

### 2.11 Regression model between experimental and simulation results

To determine the stability effect driven by heme-protein interaction, two factors were considered. The first is the interaction score determined by the weighted frequency of various interactions – carbon hydrogen bonds from the backbone (BB), hydrogen bonds, carbon hydrogen bonds, and salt bridges from the sidechain (SC) – divided by their respective average distances in Å (Equation (1)). The second is the inverse of the volume of the amino acid at the residue 181 in Å^3^ (Equation (2)). HemeLock Index for quantifying heme-protein interaction is determined as the sum of two scores (Equation (3)) and its linear relationship between *T*_m_ is formulated as Equation (4).

(1) $$\begin{array}{c} \text{Interaction}\;\text{score}=w_{1}\times \frac{\text{BB}\;\text{CH}\;I}{\text{BB}\;\text{CH}\;D}+w_{2}\times \frac{\text{SC}\;H\;I}{\text{SC}\;H\;D}+w_{3}\times \frac{\text{SC}\;\text{CH}\;I}{\text{SC}\;\text{CH}\;D}+w_{4}\times \frac{\text{SC}\;\text{SB}\;I}{\text{SC}\;\text{SB}\;D} \end{array}$$

(2) $$\begin{array}{c} \text{Amino}\;\text{acid}\;\text{size}\;\text{score}=\frac{w_{5}}{\text{Volume}\;\text{of}\;\text{amino}\;\text{acid}\;\left({Å}^{3}\right)} \end{array}$$

(3) $$\begin{array}{c} \text{HemeLock}\;\text{Index}=\text{Interaction}\;\text{score}+\text{Amino}\;\text{acid}\;\text{size}\;\text{score} \end{array}$$

(4) $$\begin{array}{c} T_{m}\left({}^{\circ}C\right)=a\times \left(\text{HemeLock}\;\text{Index}\right)+b \end{array}$$

where ‘BB’ represents the backbone, ‘SC’ is the sidechain, ‘CH’ is carbon hydrogen bond, ‘H’ is hydrogen bond, ‘SB’ is salt bridge, ‘I’ denotes interaction, ‘D’ is distance (Å), and ‘*w*’ is the weight factor. All units were ignored while calculating each score, and therefore, all of the score values have no units. A linear relationship was established between the scores of *Pc*LiP01 WT and V181X variants and their experimental *T*_m_, optimizing the weight factors for the best coefficient of determination by utilizing Excel Solver.

### 2.12 Computational tool-based mutation of PcLiP01

To generate proposed mutant designs of *Pc*LiP01 using PROSS (the Protein Repair One-Stop Shop) [33], the PDB ID of *Pc*LiP01 (1B82) was submitted. Critical residues associated with enzyme functionality were fixed to remain unaltered during the muntation process. These included the surface active site W171 [34]; N-/O-glycosylation sites N257 [35], T320 and S334 [36]; the proximal and distal histidines H176, D238 [37] and H47; as well as the long-range electron transfer pathway residues F205 [36] and W251 [37,38]. All other settings were left as default. From the nine mutant designs suggested by PROSS, the very last design was excluded. The first, fifth and the eighth designs were selected as mutation candidates (PROSS1 (S49A/A133P/E163N/T240L/V262Q); PROSS2 (G10K/M56L/P96S/I155L/N156A/N159A/S174A/S202T/S245P added to PROSS1); PROSS3 (K7Q/Q26T/A55S/D75A/A80N/G102N/A110H/T130V/T150S/L167A/A214L/E232V/H239F/S259E added to PROSS2); for detail, see Table S3). The sequence-optimized DNA for *E. coli* expression was synthesized by Macrogen (Korea) and cloned into pET-21b(+) vector using *Eco*RI and *Nde*I restriction sites.

### 2.13 Bioinformatics analysis

Structural homologs of heme enzymes were searched using Foldseek [39] by using the structure on the Protein Data Bank (www.rcsb.org) of heme enzymes as input files. From the results, AFDB-SWISSPROT containing 504 hits were selected for multiple sequence alignments and a phylogenetic tree construction. On the other hand, PDB100 list with 264 hits were checked to find results which their structures are reported. Mutants were excluded to remain 41 wildtype structures of proteins. Multiple sequence alignments were performed using ClustalW. Sequences shorter than 250 amino acids or longer than 770 amino acids were not used for alignment. Sequences with minute differences were reduced manually for the visual clarity. Evolutionary analyses were conducted in MEGA X [40]. The evolutionary history was inferred using the Neighbor-Joining method [41]. The percentage of replicate trees in which the associated taxa clustered together in the bootstrap test (1000 replicates) were calculated [42]. The optimal tree had the sum of branch length = 25.66397643. The tree was drawn to scale, with branch lengths in the same units as those of the evolutionary distances used to infer the phylogenetic tree. The evolutionary distances were computed using the JTT matrix-based method [43] and are in the units of the number of amino acid substitutions per site. The analysis involved 106 amino acid sequences. All ambiguous positions were removed for each sequence pair. There were a total of 918 positions in the final dataset.

### 2.14 Heme-protein binding energy calculation

The PDB structure files of each enzymes found by Foldseek were loaded on Discovery Studio. CHARMM force field was applied and proteins were prepared at the default condition (pH 7.4 and ionic strength 0.145 M). The binding energy was calculated by regarding proteins as the receptor and heme molecules as the ligand, at the default condition (298.15 K), by the following equation:

$${\text{Energy}}_{\text{binding}}={\text{Energy}}_{\text{complex}}-{\text{Energy}}_{\text{ligand}}-{\text{Energy}}_{\text{receptor}}\text{.}$$

## 3 Results and discussion

### 3.1 Exploring key regions for thermal stability in heme enzymes

In protein engineering, particularly regarding heme enzymes, prioritizing structural similarities over sequence comparisons is essential. This strategy effectively addresses issues associated with sequence similarity-based approaches, such as the discrepancy between sequence similarity and spatial configuration, difficulty in identifying structural nuances and interactions, and challenges in predicting dynamic protein behavior. Emphasizing structural context is crucial for accurately identifying residues critical for properties like thermal stability. By adopting a structure-based approach, we utilized structural similarities and comparative analysis to mitigate these issues.

**Fig. 1:**
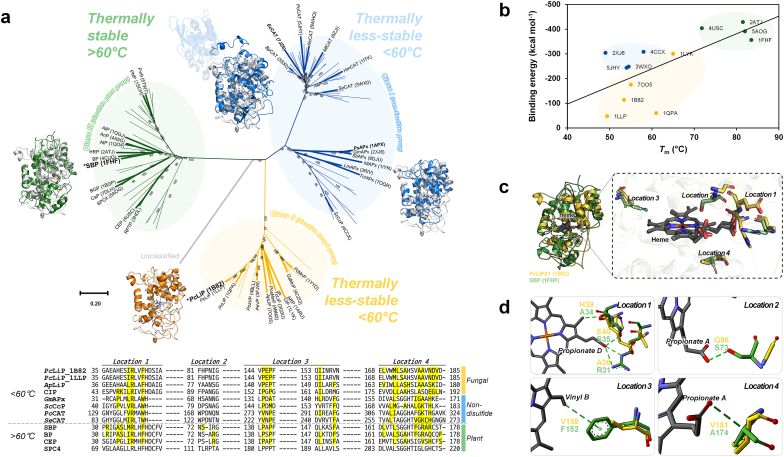
Discovery of key residues for the thermostability of heme enzymes due to heme-protein interactions. (a) Phylogenetic tree of heme enzymes based on the structural similarity suggested from Foldseek. The multiple sequence alignment at the bottom is a partial sequence alignment of the heme interacting and structurally homologous region from enzymes within enzyme groups identified through a structure-based homology search. Yellow highlights at the sequence alignments indicate the residues interacting with their heme molecules in the PDB structures. For abbreviations, see Table S4. (b) Linear relationships between *T*_m_ and heme-protein binding energy of heme enzymes. (c) The Overall overlapped structure of SBP (PDB ID: 1FHF, green) and *Pc*LiP01 (PDB ID: 1B82, orange) and detailed environment near the heme. (d) Residues showing differences in interaction with heme.

Using Foldseek [39], a recently developed tool that provides structural similarity information, we performed phylogenetic tree analysis on heme-containing enzymes (Fig. 1a). Three distinct groups were characterized by their origin and thermal stability (Table S4): the non-disulfide group (class I, with lower *T*_m_), the fungal group (class II, with lower *T*_m_), and the plant group (class III, with higher *T*_m_). The investigation into the reported *T*_m_ of these enzymes confirmed that plant-derived enzymes exhibited significantly higher thermal stability compared to the other groups. However, a simple sequence comparison was insufficient to pinpoint the specific residues that affect thermal stability. Thus, we investigated several properties that may affect *T*_m_. Interestingly, this tendency for high *T*_m_ was more strongly associated with the binding energy and interaction between heme and protein rather than disulfide bonding (Fig. 1b and Fig. S1). Consequently, these observations led us to hypothesize that the interaction between heme and its surrounding environment plays a crucial role in determining the enzyme's stability and *T*_m_.

### 3.2 Comparing structure and stability of high- and low-T_m_ heme enzymes

To investigate the impact of changes in heme coordination on thermal stability, we selected the low-*T*_m_ *Pc*LiP01 (lignin peroxidase isozyme 1 from *Phanerochaete chrysosporium*, also known as LiPH8), as an *E. coli*-expressible candidate enzyme. Compared to the structure of high-*T*_m_ soybean peroxidase (SBP; 83.5 °C) [44], *Pc*LiP01 exhibited significant differences in the residues coordinating its heme group (Fig. 1c). Specifically, residues G86, V181, V158, A36, E40, and H39 in *Pc*LiP01 lack interaction with the heme, whereas their counterparts in SBP interact with various functional groups of heme: S73, A174 (with propionate A), F152 (with vinyl B), R31, S35 (with propionate D), and A34 (with methyl D), as shown in Fig. 1d. These differences in interactions suggest potential targets for modifying thermal stability.

**Fig. 2:**
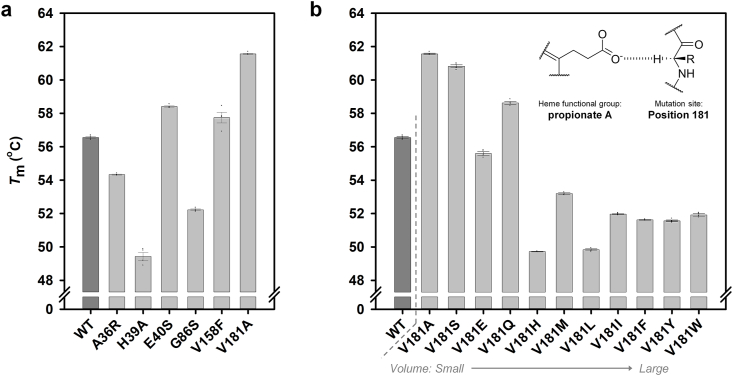
Thermodynamic stability analysis of *Pc*LiP01 by measuring the *T*_m_ of (a) homology mutants and (b) V181X saturation mutants, determined at pH 3.0. V181X mutants were ordered by their amino acid volume, from small to large. The chemical formula represents the carbon hydrogen bond between the heme propionate A group and the hydrogen of the residue 181 backbone.

For exploring the effect of each mutation, single mutations on six residues were introduced in *Pc*LiP01 (Table S1). From *T*_m_ measurement results, V181A (61.56 °C) and E40S (58.40 °C) displayed increased *T*_m_ by 5.01 °C and 1.85 °C compared to the WT, followed by V158F (57.74 °C), A36R (54.33 °C), G86S (52.22 °C) and H39A (49.43 °C) (Fig. 2a). This result suggests that position V181 is crucially associated with the thermal property of *Pc*LiP01. Further, by exploring the effect on the thermal stability of various residues at the V181 site, we could identify that the size of amino acids [45] revealed a moderate linear correlation (R^2^ = 0.645, Fig. S2), in which smaller amino acids showed increased *T*_m_ values as described in Fig. 2b (additional *T*_m_ data of all V181X mutants are available in Table S5). The melting curves for these mutants are shown in Fig. S3. However, despite this apparent correlation, a more detail analysis is warranted to fully elucidate how these mutations influence the thermal stability of the enzyme.

### 3.3 Thermodynamic stability and kinetic property of PcLiP01 WT, single and double mutants

**Fig. 3:**
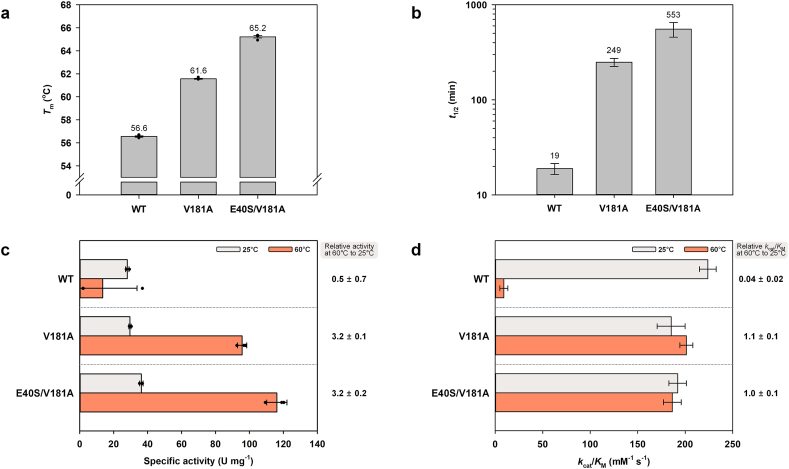
*T*_m_ (a), *t*_1/2_ under the heat inactivation at 55 °C (b), specific activity at 25 and 60 °C (c), and catalytic efficiency (*k*_cat_/*K*_M_) at 25 and 60 °C (d) of *Pc*LiP01 WT, V181A and E40S/V181A.

Next, we constructed the double mutation E40S/V181A by combining two single mutations that exhibited the highest increase in *T*_m_. As shown in Fig. 3a, E40S/V181A variant exhibited a notable increase in *T*_m_ at pH 3.0, reaching 65.21 °C, which is 8.66 °C higher than the WT. Melting curves for these mutants are available in Fig. S4. Its thermal stability, assessed via heat inactivation at 55 °C, showed a remarkable 29-fold improvement over the WT, with a half-life of 553 min (Fig. 3b). The specific activity at 25 °C was slightly higher than that of the WT (Fig. 3c), and a slight decrease in catalytic efficiency (*k*_cat_/*K*_M_) was observed compared to the WT (Fig. 3d). However, this reduction is marginal when weighed against the substantial gain in stability. Additionally, *k*_cat_/*K*_M_ of *Pc*LiP01 V181A and E40S/V181A at 60 °C was still maintained, with 3.2-fold increased specific activity, compared with values obtained at 25 °C (Fig. 3c and d).

Saturation curves, and *k*_cat_, *K*_M_ at 25 °C and 60 °C, and *k*_d_ at 55 °C for these mutants are also presented in Fig. S5 and Table S6. These results imply that introduced mutations increased stability but showed a negligible trade-off in catalytic efficiency and that the slightly reduced catalytic efficiency was well maintained even at high temperatures. Enzyme mutations aimed at stabilization often negatively impact function [46,47], and vice versa [48,49]. Previous works on *Pc*LiP01 also demonstrated this trade-off with an improvement in thermal stability accompanied by a reduction in catalytic efficiency (with an increased *T*_m_ of 0.7–2.8 °C and a decreased *k*_cat_/*K*_M_ of 34–36 %) [12,13]. Given this typical experience, focusing mutation strategies on heme-protein interactions could represent a viable approach to enzyme engineering, one that does not excessively compromise enzyme function.

### 3.4 MD simulation of PcLiP WT and V181X and its analysis

**Fig. 4:**
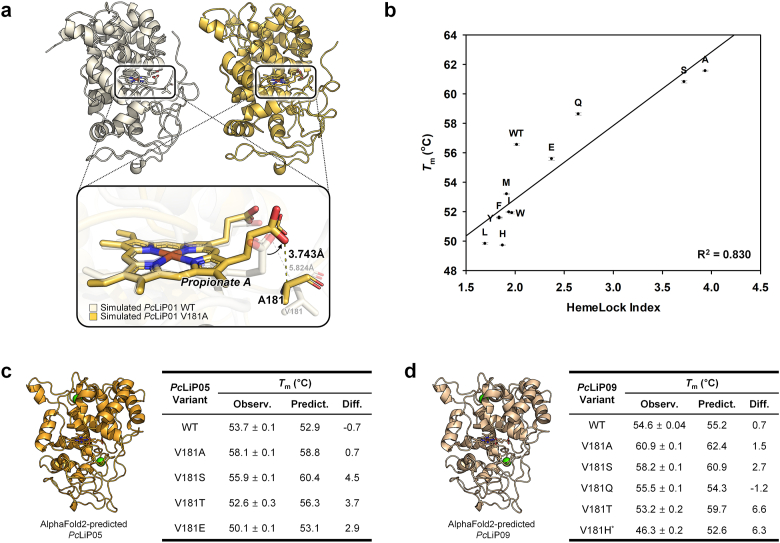
(a) Structural differences of *Pc*LiP01 WT (ivory) and V181A (yellow) in a 200 ns snapshot from MD simulation, highlighting heme propionate A and residue 181, with their oxygen-Cα distances. (b) *T*_m_ correlation with HemeLock Index for *Pc*LiP01 WT and V181X variants. (c–d) AlphaFold2-predicted enzyme structures, experimentally observed *T*_m_ (Observ.), predicted *T*_m_ by HemeLock Index (Predict.) and their difference (Diff.) for the WT and V181X variants of *Pc*LiP05 (c) and *Pc*LiP09 (d). Asterisk (*) indicates the variant without activity.

To elucidate the factor affecting the thermal stability of *Pc*LiP01 variants, MD simulations were conducted under their *T*_m_ measurement condition (pH 3) and room temperature (25 °C) for *Pc*LiP01 WT and V181A. To find differences between WT and V181A, we first checked the distance between heme and residue 181 in a 200 ns snapshot (Fig. 4a). In contrast to the WT, where residue 181 is farther from heme propionate A (distance: 5.824 Å), in the V181A mutant, A181 is positioned closer, forming a carbon hydrogen bond with heme propionate A (distance: 3.743 Å). To see whether differences in the motion of the heme-protein complex constantly remain even in dynamic conditions, we analyzed MD simulation results up to 500 ns. It was revealed that the ligand complex movement is less in V181A than in WT, with distances from residue 181 to heme (100–500 ns simulation) being 6.02 ± 0.25 Å for WT and 3.50 ± 0.20 Å for V181A. These results imply the distance difference and following interaction difference between heme-residue 181 may affect their stability changes.

We further compared the structural changes to determine the overall fluctuation. As a result, V181A had less RMSD than WT, showing 2.48 ± 0.31 Å for WT and 1.99 ± 0.12 Å for V181A (Fig. S6a). Especially at the higher temperature of 80 °C, heme-residue 181 distance and RMSD of WT greatly fluctuated, whereas those of V181A remained relatively stable throughout the 100–500 ns simulation. The distance from residue 181 to heme was 5.80 ± 2.93 Å for WT and 7.02 ± 0.88 Å for V181A; RMSD values were 6.20 ± 1.77 Å for WT and 3.74 ± 0.31 Å for V181A (Fig. S6b). Several studies have reported a correlation between decreased RMSD or distances and increased protein stability [50,51]. Lower RMSD or distance changes reveal enhanced protein stability by showing minimal flexibility and deviation from the native state, indicating that the MD simulation provides a precise understanding of the stability change. Thus, we concluded that the enhanced thermal stability of the mutant is due to its reduced flexibility and closer heme-residue interaction.

### 3.5 HemeLock index model for cofactor-heme enzyme interaction

To establish a model for accurate estimation of *T*_m_ of *Pc*LiP01, a heme-containing enzyme, we devised new equations based on key factors for the interaction between heme and residue 181: the frequency of non-bond interactions, and their distance (Equations (1), (2), (3), (4))). The values of these factors are shown in Table S7. Consequently, the HemeLock Index (Equations (5), (6))), indicating stability influenced by heme and its surrounding residues, was suggested and well-correlated with *T*_m_ (R^2^ = 0.830) as shown in Fig. 4b. Compared to the previous simple correlation between *T*_m_ and amino acid size in section 3.2 (R^2^ = 0.645, Fig. S2), the accuracy of the suggested model has markedly improved. This enhancement highlights the importance of incorporating interactions and distances between heme and protein, observed over time through MD simulations, into predictions of protein stability. Moreover, the distribution of each score in the HemeLock Index model indicates that amino acid size plays a more substantial role in *T*_m_ than interactions and distance which are affected by the charge or polarity of residues (backbone: 0–0.750; sidechain: 0–0.348; volume of amino acid: 1.239–3.186).

(5) $$\begin{array}{c} \text{HemeLock}\;\text{Index}=\\ 2.974\times \frac{\text{BB}\;\text{CH}\;I}{\text{BB}\;\text{CH}\;D}+0.9201\times \frac{\text{SC}\;H\;I}{\text{SC}\;H\;D}+\frac{282.3}{\text{Volume}\;\text{of}\;\text{amino}\;\text{acid}} \end{array}$$

(6) $$\begin{array}{c} T_{m}\left({}^{\circ}C\right)=4.995\times \left(\text{HemeLock}\;\text{Index}\right)+42.86 \end{array}$$

where ‘BB’ stands for backbone, ‘SC’ for sidechain, ‘CH’ for carbon hydrogen bond, ‘H’ for hydrogen bond, ‘I’ for interaction, and ‘D’ for distance.

Among the isozymes of *Pc*LiPs, *Pc*LiP05 and *Pc*LiP09 possess relatively lower *T*_m_ and share the Valine residue at position 181 with *Pc*LiP01. Due to this commonality, we further applied the proposed model to the V181X mutation simulations of *Pc*LiP05 and *Pc*LiP09, aiming to verify the accuracy of the model as well as to enhance the interaction between residue 181 and heme, thereby potentially increasing their stability. Experimental *T*_m_ of mutants with various predicted *T*_m_ were checked. The model anticipated that the V181A and V181S mutations in *Pc*LiP05 and *Pc*LiP09 would exhibit the highest *T*_m_ increases, similar to those observed in *Pc*LiP01. The experimental results confirmed these predictions, with both mutations achieving the highest *T*_m_ values as estimated. Furthermore, *T*_m_ values for other mutations aligned closely with the model's predictions (Fig. 4c and d). Their melting curves and additional *T*_m_ data are in Fig. S7 and Table S8. These outcomes demonstrate that applying the HemeLock Index to highly homologous *Pc*LiP isozymes is viable, highlighting the model's utility across similar enzyme systems.

We additionally tried to adjust the weight factor for amino acid volume (*w*_5_) in our model to address the larger-than-expected discrepancies between predicted and experimental *T*_m_ values, particularly the 6.6 °C difference observed for *Pc*LiP09's V181T variant. This adjustment (*w*_5_ = 233.1 for *Pc*LiP05; *w*_5_ = 223.3 for *Pc*LiP09) improved prediction accuracy, reducing the mean squared error in *T*_m_ values from 8.7 to 3.5 for *Pc*LiP05 and from 15.8 to 9.2 for *Pc*LiP09. These findings suggest that while the overall approach is broadly applicable, certain parameters may require fine-tuning depending on the specific protein. Future refinement and validation with a larger experimental dataset could further enhance the robustness of our model.

### 3.6 Comparison of the proposed mutation strategy with PROSS

To explore the potential of synergistic effects between our mutation strategy and PROSS, a widely acclaimed computational tool for designing stable protein mutants [33], we compared the specific activity and *T*_m_ of our constructed mutants with those predicted by PROSS (Table 1). Among the three mutants, PROSS3 could not be successfully purified, likely due to an excessive number of mutations (29 out of 351 amino acids). In contrast, PROSS1, which incorporates five mutations, demonstrated a 3.46 °C increase in *T*_m_. Melting curves of these mutants are detailed in Fig. S8. These results suggest that our strategy effectively captures the unique characteristics of heme enzymes, enhancing their prediction and thermal stability. Since PROSS mutations do not involve heme-interacting residues, combining them with E40S/V181A could yield synergistic effects, and further study is needed to validate this hypothesis.

**Table 1:** Specific activity and *T*_m_ of *Pc*LiP01 variants constructed by the proposed strategy and PROSS.

Variants	WT	The proposed approach		PROSS suggestions	
		V181A	E40S/V181A	PROSS1	PROSS2
**Number of mutations**	–	1	2	5	14
**Specific activity (U mg**^**−**^**^1^)**	28.1 ± 1.0	29.6 ± 0.6	36.3 ± 1.0	28.6 ± 0.6	36.1 ± 0.2
***T***_**m**_**(°C)**	56.55 ± 0.06	61.56 ± 0.04	65.21 ± 0.10	60.01 ± 0.07	56.19 ± 0.36

### 3.7 Enhanced milled wood lignin (MWL) depolymerization by PcLiP01 mutants

**Fig. 5:**
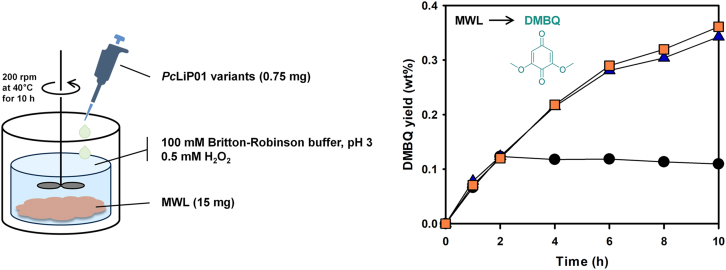
Schematic diagram of enzymatic MWL depolymerization and DMBQ production reaction using *Pc*LiP01 variants at 40 °C, and DMBQ yield by *Pc*LiP01 WT (black circle), V181A (blue triangle), and E40S/V181A (orange square) by time. (For interpretation of the references to colour in this figure legend, the reader is referred to the Web version of this article.)

Milled wood lignin (MWL), which retains the native structure of biomass-derived lignin [52], was the substrate used to assess the depolymerization efficacy of *Pc*LiP01 WT and its mutants at an elevated temperature (Fig. S9). Enzymatic conversion reactions were conducted at 40 °C, a temperature strategically selected to optimize enzyme activity and stability, emphasizing the thermal resistance of the E40S/V181A mutant. The depolymerization progress was monitored over a 10-h period by quantifying the yield of 2,6-dimethoxy-1,4-benzoquinone (DMBQ), a direct product of MWL degradation. The WT enzyme exhibited a rapid decrease in activity, halting DMBQ production at the 2-h mark with a yield of 0.12 %. In contrast, the E40S/V181A mutant continued to produce DMBQ consistently throughout the 10-h experiment, achieving a yield of 0.36 % DMBQ, which represents a threefold increase compared to WT (Fig. 5). These results suggest the E40S/V181A mutant's superior capability to maintain activity and enhance MWL conversion efficiency at temperatures that inactivate the WT enzyme. The robust performance of the mutant at 40 °C indicates its potential for industrial lignin valorization, offering a sustainable solution for biomass processing.

Several reports have shown that heme-protein interactions significantly impact enzyme stability. For instance, the *T*_m_ difference between holo- and apo-forms of SBP and cytochrome *b*_5_ is as much as 45.5 °C and 41.5 °C [44,53]. Also, *T*_m_ of apo-*Pc*LiP01 was found to be below 25 °C (Fig. S10). These indicate the critical role of heme-protein interaction in the stability of heme proteins.

In the article, we have presented a strategic ‘keystone cofactor heme-interaction approach’ for increasing *T*_m_, fine-tuning the interactions between the heme cofactor—a critical cofactor for enzyme stability—and its surrounding residues. Using this approach, we obtained mutants with enhanced thermal stability without any functional trade-offs, as confirmed through MD simulations. It was also possible to build a predictive model with a good correlation with *T*_m_ applicable to both *Pc*LiP01 and its isozymes. Additionally, these thermostable *Pc*LiP01 mutants showed higher conversion of MWL than WT at an elevated temperature.

Previous studies on *Pc*LiP have reported limited *T*_m_ increases of 1.5 °C, 2.8 °C, and 1.8 °C by adding salt bridges, disulfide bridges, and ancestral mutations, respectively [[12], [13], [14]]. In contrast, our method has shown a significant increase of *T*_m_, underscoring the potential of heme-protein interactions to enhance the thermal stability of heme-containing enzymes. As far as we know, this is the first report to successfully enhance thermal stability in heme enzymes by specifically focusing on these interactions and quantifying these effects.

This study emphasizes the importance of cofactors in protein stabilization, which is often overlooked, and also highlights the potential for enzymatic lignin conversion in high-temperature environments, which are commonly required in industrial processes. For an in-depth understanding of the stability of heme or other cofactor-containing proteins, customized analyses for each protein and structural analysis through X-ray crystallography would be valuable.

## 4 Conclusions

Our approach, based on structural similarity, allowed for straightforward identification of important sites for protein stability. Reliant on this, we engineered LiP variants showing selectively enhanced thermal stability without *k*_cat_ change by mutating residues around heme. Further, by applying MD simulation of mutants that induce changes in near-heme coordination and combining this with experimental data, we established a precise *T*_m_ prediction model. Finally, enzymatic depolymerization of MWL showed that the suggested mutants had higher conversion than WT at a high temperature. We expect this study to be helpful in the rational design of enzymes and deeper comprehension of cofactor dynamics.

## Funding

This work was supported by the National Research Foundation grants [2020R1A5A1019631, 2022M3J1A1052840, RS-2024-00396026].

## Data and code availability

Data will be made available on request.

## CRediT authorship contribution statement

**Joo Yeong Park:** Writing – original draft, Validation, Investigation, Conceptualization. **Seunghyun Han:** Writing – original draft, Validation, Investigation, Formal analysis. **Doa Kim:** Writing – original draft, Validation, Investigation, Formal analysis. **Trang Vu Thien Nguyen:** Validation, Investigation. **Youhyun Nam:** Writing – original draft, Validation, Investigation, Formal analysis, Conceptualization. **Suk Min Kim:** Writing – review & editing, Supervision, Conceptualization. **Rakwoo Chang:** Writing – review & editing, Supervision, Conceptualization. **Yong Hwan Kim:** Writing – review & editing, Supervision, Conceptualization.

## Declaration of competing interest

The authors declare that they have no known competing financial interests or personal relationships that could have appeared to influence the work reported in this paper.
