# Supplementary material for: Enhancing the thermostability of lignin peroxidase: Heme as a keystone cofactor driving stability changes in heme enzymes
Source: Heliyon. 2024 Aug 30;10(17):e37235. doi: 10.1016/j.heliyon.2024.e37235 (PMC11419925; doi:10.1016/j.heliyon.2024.e37235)
Supplement: Multimedia component 1 [file mmc1.docx]

Supporting Information

**Enhancing the thermostability of lignin peroxidase: heme as a keystone cofactor driving stability changes in heme enzymes**

*Joo Yeong Park^a,1^, Seunghyun Han^a,1^, Doa Kim^a,1^, Trang Vu Thien Nguyen^a^, Youhyun Nam^b^, Suk Min Kim^a^, Rakwoo Chang^b^ and Yong Hwan Kim^a,c,*^*

^a^ School of Energy and Chemical Engineering, Ulsan National Institute of Science and Technology (UNIST), 50, UNIST-gil, Ulsan 44919, Republic of Korea

^b^ Department of Applied Chemistry, University of Seoul, 163, Seoulsiripdae-ro, Seoul 02504, Republic of Korea

^c^ Graduate School of Carbon Neutrality, Ulsan National Institute of Science and Technology (UNIST), 50, UNIST-gil, Ulsan 44919, Republic of Korea

^*^ Corresponding authors. E-mail address: smkimlife@unist.ac.kr (S. M. Kim), rchang90@uos.ac.kr (R. Chang), metalkim@unist.ac.kr (Y. H. Kim)

^1^ Co-first authors.

**Table S1.** List and sequence of primers used for the site direct mutagenesis of *Pc*LiPs.

| **Protein** | **Mutation** | **Forward primer (5’→3’)** | **Reverse primer (5’→3’)** |
| --- | --- | --- | --- |
| *Pc*LiP01 | A36R | CAATGCGGTCGTGAAGCTCATGAAAGTATCCGT | ATGAGCTTCACGACCGCATTGACCGCCA |
|  | H39A | GCAGAAGCTGCGGAAAGTATCCGTCTGGTT | GATACTTTCCGCAGCTTCTGCACCGCATTG |
|  | E40S | GAAGCTCATTCTAGTATCCGTCTGGTTTTCCACGA | ACGGATACTAGAATGAGCTTCTGCACCGCATT |
|  | G86S | CCGAATATTTCTCTGGATGAAATCGTTAAACTG | GATTTCATCCAGAGAAATATTCGGATGAAAAGC |
|  | V158F | ATCAACCGCTTTAATGATGCAGGCGAATTT | TGCATCATTAAAGCGGTTGATAATCTGGTC |
|  | V181A | GTGGCAGCAGCAAACGATGTCGACCCGACC | GACATCGTTTGCTGCTGCCACAGAGTGTGCGCT |
|  | V181C | GTGGCAGCATGCAACGATGTCGACCCGACC | GACATCGTTGCATGCTGCCACAGAGTGTGCG |
|  | V181D | GTGGCAGCAGATAACGATGTCGAC | GACATCGTTATCTGCTGCCACAGAGTGTG |
|  | V181E | GTGGCAGCAGAAAACGATGTCGACCCGACC | GGGTCGACATCGTTTTCTGCTGCCACAGAGTGTGC |
|  | V181F | GTGGCAGCATTTAACGATGTCGACCC | ACATCGTTAAATGCTGCCACAGAGT |
|  | V181G | GTGGCAGCAGGCAACGATGTCGACCCGACC | GGGTCGACATCGTTGCCTGCTGCCACAGAGTGTGC |
|  | V181H | GTGGCAGCACATAACGATGTCGACCCG | GACATCGTTATGTGCTGCCACAGAGTGTGCG |
|  | V181I | GTGGCAGCAATTAACGATGTCGACCCGACC | GGTCGACATCGTTAATTGCTGCCACAGAGTGTGC |
|  | V181K | GTGGCAGCAAAAAACGATGTCGACCCGACC | GGTCGACATCGTTTTTTGCTGCCACAGAGTGTGC |
|  | V181L | GTGGCAGCACTGAACGATGTCGACCCGACC | GGGTCGACATCGTTCAGTGCTGCCACAGAGTGTGC |
|  | V181M | TGGCAGCAATGAACGATGTCGACCCG | CGACATCGTTCATTGCTGCCACAGAGTGTG |
|  | V181N | GTGGCAGCAAACAACGATGTCGACCCGAC | GACATCGTTGTTTGCTGCCACAGAGTGTGCG |
|  | V181P | GTGGCAGCACCGAACGATGTCGACCCGACC | GACATCGTTGGCTGCTGCCACAGAGTGTGC |
|  | V181Q | GTGGCAGCACAGAACGATGTCGACCCGAC | GACATCGTTCTGTGCTGCCACAGAGTGTGCG |
|  | V181R | GTGGCAGCACGTAACGATGTCGACCCGACC | GGGTCGACATCGTTACGTGCTGCCACAGAGTGTGC |
|  | V181S | GTGGCAGCAAGCAACGATGTCGACCCGACC | GACATCGTTGCTTGCTGCCACAGAGTGTGCG |
|  | V181T | GTGGCAGCAACCAACGATGTCGACCCGAC | GACATCGTTGGTTGCTGCCACAGAGTGTGC |
|  | V181W | GTGGCAGCATGGAACGATGTCGACCCGACC | GACATCGTTCCATGCTGCCACAGAGTGTGCG |
|  | V181Y | GTGGCAGCATATAACGATGTCGACCCG | GACATCGTTATATGCTGCCACAGAGTGTGCG |
| *Pc*LiP05 | V181A | GTGGCGGCGGCAAACGATGTGGATCCGACCGTG | ACATCGTTTGCCGCCGCCACGCTATGCGCG |
|  | V181E | GTGGCGGCGGAAAACGATGTGGATCCGACCGTG | CACATCGTTTTCCGCCGCCACGCTATGCGCG |
|  | V181H | GTGGCGGCGCATAACGATGTGGATCCGACCGTG | ACATCGTTATGCGCCGCCACGCTATGCGCG |
|  | V181S | GTGGCGGCGTCTAACGATGTGGATCCGACCGTG | CATCGTTAGACGCCGCCACGCTATGCGCG |
|  | V181T | GTGGCGGCGACAAACGATGTGGATCCGACCGTG | CATCGTTTGTCGCCGCCACGCTATGCGC |
| *Pc*LiP09 | V181A | GTGGCGGCGGCAAACGATGTGGATCCGACCATT | CATCGTTTGCCGCCGCCACGCTATGCGCG |
|  | V181E | GTGGCGGCGGAAAACGATGTGGATCCGACCATT | CATCGTTTTCCGCCGCCACGCTATGCGCG |
|  | V181H | GTGGCGGCGCATAACGATGTGGATCCGACCATT | ACATCGTTATGCGCCGCCACGCTATGCGCG |
|  | V181Q | GTGGCGGCGCAAAACGATGTGGATCCGACCATT | ACATCGTTTTGCGCCGCCACGCTATGCGCG |
|  | V181R | GTGGCGGCGCGTAACGATGTGGATCCGACCATT | CATCGTTACGCGCCGCCACGCTATGCGCG |
|  | V181S | GTGGCGGCGTCTAACGATGTGGATCCGACCATT | CATCGTTAGACGCCGCCACGCTATGCGCG |
|  | V181T | GTGGCGGCGACAAACGATGTGGATCCGACCATT | CATCGTTTGTCGCCGCCACGCTATGCGC |
|  | V181Y | GTGGCGGCGTATAACGATGTGGATCCGACCATT | CATCGTTATACGCCGCCACGCTATGCGCG |

**Table S2.** Possible hydrogen donor atoms of each amino acid for sidechain-propionate group hydrogen bonds and carbon hydrogen bonds formation.

| **Amino acid** | **Hydrogen donor(s) for hydrogen bonds** | **Hydrogen donor(s) for carbon hydrogen bonds** |
| --- | --- | --- |
| **Arg** | NE, NH1, NH2 | CD |
| **Asn** | ND2 | - |
| **Asp** | OD2 | - |
| **Cys** | SG | - |
| **Gln** | NE2 | - |
| **Glu** | OE2 | - |
| **His** | ND1 | CD2, CE1 |
| **Lys** | NZ | CE |
| **Pro** | - | CD |
| **Ser** | OG | CB |
| **Thr** | OG1 | CB |
| **Trp** | NE1 | CD1 |
| **Tyr** | OH | - |

Abbreviations. CB, beta carbon; CD, delta carbon; CE, epsilon carbon; ND, delta nitrogen; NE, epsilon nitrogen; NZ, zeta nitrogen; NH, eta nitrogen; OG, gamma oxygen; OD, delta oxygen; OE, epsilon oxygen; OH, eta oxygen; SG, gamma sulfur [1].

**Table S3.** Mutations of *Pc*LiP01 suggested by PROSS. The PROSS2 mutant carries the PROSS1 mutations, and PROSS3 includes mutations from both PROSS1 and PROSS2.

| **Mutant** | **List of mutations and their locations on the secondary structure** | | |
| --- | --- | --- | --- |
|  | **α-helix** | **β-sheet** | **Loop** |
| **PROSS1** | S49A/E163N/T240L/V262Q | - | A133P |
| **PROSS2** | M56L/P96S/I155L/N156A/N159A/S174A | - | G10K/S202T/S245P |
| **PROSS3** | Q26T/A55S/A110H/L167A/H239F/S259E | T130V | K7Q/D75A/A80N/G102N/T150S/A214L/E232V |

**Table S4.** List of structural homologs of heme enzymes from Foldseek and their reported *T*_m_, calculated binding energy, existing disulfide bridge, and heme-protein hydrogen bonds.

| **Group (class^†^)** | **Enzyme (EC No.)** | **Origin** | **Name (UniProt ID)** | **PDB ID** | ***T*_m_, ℃ (source)** | **Binding energy^‡^ (kcal mol^-1^)** | **Disulfide bridges** | **Hydro-gen bond** | **Carbon hydrogen bond** | **Electro-static** | **Hydro-phobic** |
| --- | --- | --- | --- | --- | --- | --- | --- | --- | --- | --- | --- |
| Non-disulfide group (Class I) | Ascorbate peroxidase (APx, 1.11.1.11) | *Glycine max* | *Gm*APx (Q43758) | 2XJ6 | 49 [2] | -303.9 | 0 | 5 | 3 | 3 | 25 |
|  |  | *Pisum sativum* | *Ps*APx (P48534) | 1APX | N.D. | – | 0 | – | – | – | – |
|  |  | *Leishmania major* | *Lm*APx (Q4Q3K2) | 3RIV | N.D. | – | 0 | – | – | – | – |
|  |  | *Nicotiana tabacum* | *Nt*APx (Q8LNY5) | 1IYN | N.D. | – | 0 | – | – | – | – |
|  |  | *Sorghum bicolor* | *Sb*APx (C5WNL8) | 8DJU | N.D. | – | 0 | – | – | – | – |
|  |  | *Trypanosoma cruzi* | *Tcr*APx (Q8I1N3) | 7OPT, 7OQR | N.D. | – | 0 | – | – | – | – |
|  | Catalase-peroxidase (CAT, 1.11.1.21) | *Burkholderia pseudomallei* | *Bp*CAT (Q3JNW6) | 5SX0 | N.D. | – | 0 | – | – | – | – |
|  |  | *Escherichia coli* | *Ec*CAT (A0A037YMJ1) | 7JZ6 | N.D. | – | 0 | – | – | – | – |
|  |  | *Mycobacterium tuberculosis* | *Mt*CAT (P9WIE5) | 7AG8 | N.D. | – | 0 | – | – | – | – |
|  |  | *Pyricularia oryzae* | *Po*CAT (A4QUT2) | 5JHY | 54 [3] | -243.8 | 1 | 5 | 2 | 1 | 21 |
|  |  | *Synechococcus elongatus* | *Se*CAT (Q31MN3) | 3WXO | 54.5 [4] | -248.5 | 0 | 5 | 2 | 1 | 25 |
|  |  | *Haloarcula marismortui* | *Hm*CAT (O59651) | 1ITK | N.D. | – | 0 | – | – | – | – |
|  |  | *Mycobacterium tuberculosis* | *Mt*CAT (A0A0D5ZBI4) | 6ZJI | N.D. | – | 0 | – | – | – | – |
|  |  | *Neurospora crassa* | *Nc*CAT (Q8X182) | 5WHS, 5WHQ | N.D. | – | 0 | – | – | – | – |
|  | Cytochrome *c* peroxidase (CcP, 1.11.1.5) | *Saccharomyces cerevisiae* | *Sc*CcP (P00431) | 4CCX | 58 [5] | -308.3 | 0 | 5 | 2 | 1 | 26 |
| Fungal group (Class II) | Arthromyces ramosus peroxidase (ARP, 1.11.1.7) | *Arthromyces ramosus* | ARP (P28313) | 1ARU | N.D. | – | 4 | – | – | – | – |
|  | Coprinus cinereus peroxidase (CIP, 1.11.1.7) | *Coprinopsis cinerea* | CIP (P28314) | 1LYK | 65 [6] | -300.2 | 4 | 5 | 1 | 3 | 21 |
|  | Lignin peroxidase (LiP, 1.11.1.14) | *Agrocybe pediades* | *Ap*LiP (A0A8X6EH08) | 7OO5 | 55 [7] | -175.4 | 4 | 3 | 1 | 2 | 24 |
|  |  | *Phanerochaete chrysosporium* | *Pc*LiP (P06181) | 1B82 | 53.4 [8] | -113.8 | 4 | 4 | 0 | 0 | 21 |
|  |  | *Phanerochaete chrysosporium* | *Pc*LiP (P11542) | 1QPA | 61.0 [8] | -60.3 | 4 | 4 | 0 | 0 | 24 |
|  |  | *Phanerochaete chrysosporium* | *Pc*LiP (P49012) | 1LLP | 49.4 [8] | -47.5 | 4 | 4 | 0 | 0 | 21 |
|  |  | *Trametopsis cervina* | *Tc*LiP (Q60FD2) | 3Q3U | N.D. | – | 4 | – | – | – | – |
|  | Manganese peroxidase (MnP, 1.11.1.13) | *Phanerochaete chrysosporium* | *Pc*MnP (Q02567) | 1YYD | N.D. | – | 5 | – | – | – | – |
|  |  | *Gelatoporia subvermispora* | *Gs*MnP (M2REM6) | 4CZQ | N.D. | – | 4 | – | – | – | – |
|  |  | *Pleurotus ostreatus* | *Pos*MnP (W5IDB6) | 4BM2 | N.D. | – | 4 | – | – | – | – |
|  | Versatile peroxidase (VP, 1.11.1.16) | *Pleurotus eryngii* | *Pe*VP (O94753) | 3FJW | N.D. | – | 4 | – | – | – | – |
|  |  | *Pleurotus ostreatus* | *Pos*VP (Q9UR19) | 4BLL | N.D. | – | 4 | – | – | – | – |
| Plant group (Class III) | Anionic peroxidase (AnP, 1.11.1.7) | *Raphanus sativus* | AnP (K7N5L9) | 4A5G | N.D. | – | 4 | – | – | – | – |
|  | Arabidopsis thaliana peroxidase (AtP, 1.11.1.7) | *Arabidopsis thaliana* | AtP (Q39034) | 1QGJ | N.D. | – | 4 | – | – | – | – |
|  |  | *Arabidopsis thaliana* | AtP (Q42578) | 1QO4 | N.D. | – | 4 | – | – | – | – |
|  | Banyan peroxidase (BP, 1.11.1.7) | *Ficus benghalensis* | BP (A0A087WNH2) | 4CUO | N.D. | – | 4 | – | – | – | – |
|  | Barley grain peroxidase  (BGP, 1.11.1.7) | *Hordeum vulgare* | BGP (Q40069) | 1BGP | N.D. | – | 4 | – | – | – | – |
|  | Cationic peroxidase (CaP, 1.11.1.7) | *Panicum miliaceum* | CaP (A0A3L6SKP5) | 7DLH | N.D. | – | 4 | – | – | – | – |
|  | Chamaerops excelsa peroxidase (CEP, 1.11.1.7) | *Trachycarpus fortunei* | CEP (A0A0A0Y4H8) | 4USC | 71.8 [9] | -404 | 4 | 6 | 0 | 2 | 24 |
|  | Horseradish peroxidase  (HRP, 1.11.1.7) | *Armoracia rusticana* | HRP (P00433) | 2ATJ | 81.5 [6] | -428.8 | 4 | 6 | 2 | 3 | 21 |
|  | Major sorghum cationic peroxidase (SPC4, 1.11.1.7) | *Sorghum bicolor* | SPC4 (P84516) | 5AOG | 82 [10] | -391.3 | 4 | 7 | 1 | 4 | 19 |
|  | Peanut peroxidase (PNP, 1.11.1.7) | *Arachis hypogaea* | PNP (P22195) | 1SCH | N.D. | – | 4 | – | – | – | – |
|  | Peroxidase from Panicum virgatum (Pvi9, 1.11.1.7) | *Panicum virgatum* | Pvi9 (A0A1S4NYF8) | 5TWT | N.D. | – | 4 | – | – | – | – |
|  | Royal palm tree peroxidase (RPTP, 1.11.1.7) | *Roystonea regia* | RPTP (D1MPT2) | 3HDL | N.D. | – | 4 | – | – | – | – |
|  | Soybean peroxidase (SBP, 1.11.1.7) | *Glycine max* | SBP (O22443) | 1FHF | 83.5 [11] | -356.2 | 4 | 5 | 1 | 4 | 18 |
| Unclassi-fied | Pseudoperoxidase (PP, -) | *Leishmania major* | *Lm*PP (Q4QC30) | 5VIA | N.D. | – | 0 | – | – | – | – |

† Class corresponds to the classification method of heme peroxidases.

‡ Binding energy was calculated using Discovery Studio software (detailed in the Experiments section).

N.D.: Not determined.

–: Not checked.

Abbreviations. *Ap*, *Agrocybe pediades*; *Bp*, *Burkholderia pseudomallei*; *Ec*, *Escherichia coli*; *Gm*, *Glycine max*; *Gs*, *Gelatoporia subvermispora*; *Hm*, *Haloarcula marismortui*; *Lm*, *Leishmania major*; *Mt*, *Mycobacterium tuberculosis*; *Nc*, *Neurospora crassa*; *Nt*, *Nicotiana tabacum*; *Pc*, *Phanerochaete chrysosporium*; *Pe*, *Pleurotus eryngii*; *Po*, *Pyricularia oryzae*; *Pos*, *Pleurotus ostreatus*; *Ps*, *Pisum sativum*; *Sb*, *Sorghum bicolor*; *Sc*, *Saccharomyces cerevisiae*; *Se*, *Synechococcus elongatus*; *Tc*, *Trametes cervina*; *Tcr*, *Trypanosoma cruzi*; AnP, anionic peroxidase; APx, ascorbate peroxidase; ARP, *Arthromyces ramosus* peroxidase; AtP, *Arabidopsis thaliana* peroxidase; BGP, barley grain peroxidase; BP, banyan peroxidase; CaP, cationic peroxidase; CAT, catalase-peroxidase; CcP, cytochrome *c* peroxidase; CEP, *Chamaerops excelsa* peroxidase; CIP, *Coprinus cinereus* peroxidase; HRP, horseradish peroxidase; LiP, lignin peroxidase; MnP, manganese peroxidase; PNP, peanut peroxidase; PP, pseudoperoxidase; Pvi9, peroxidase from *Panicum virgatum*; RPTP, royal palm tree peroxidase; SBP, soybean peroxidase; SPC4, major sorghum cationic peroxidase; VP, versatile peroxidase.

**Table S5.** RZ value and *T*_m_ of *Pc*LiP01 wild type and mutants.

| **Variant** | | | **RZ** | ***T*_m_ (℃)** |
| --- | --- | --- | --- | --- |
| WT | | | 2.7 | 56.6 ± 0.1 |
| Homology mutants | | A36R | 2.6 | 54.3 ± 0.03 |
|  |  | H39A | 1.4 | 49.4 ± 0.2 |
|  |  | E40S | 3.1 | 58.4 ± 0.1 |
|  |  | G86S | 2.2 | 52.2 ± 0.1 |
|  |  | V158F | 1.0 | 57.7 ± 0.3 |
| V181X variants | RZ > 2.5 | V181A | 3.2 | 61.6 ± 0.04 |
|  |  | V181E | 2.8 | 55.6 ± 0.1 |
|  |  | V181F | 3.2 | 51.6 ± 0.02 |
|  |  | V181H | 2.6 | 49.7 ± 0.02 |
|  |  | V181I | 2.8 | 52.0 ± 0.03 |
|  |  | V181L | 2.8 | 49.8 ± 0.1 |
|  |  | V181M | 2.9 | 53.2 ± 0.04 |
|  |  | V181Q | 2.7 | 58.6 ± 0.1 |
|  |  | V181S | 2.9 | 60.8 ± 0.1 |
|  |  | V181W | 2.9 | 51.9 ± 0.1 |
|  |  | V181Y | 2.7 | 51.6 ± 0.1 |
|  | RZ < 2.5 | V181C | 1.8 | 49.5 ± 0.3 |
|  |  | V181D | 2.1 | 49.8 ± 0.04 |
|  |  | V181G | 2.2 | 56.1 ± 0.1 |
|  |  | V181K | 2.49 | 51.2 ± 0.1 |
|  |  | V181N | 2.1 | 51.3 ± 0.04 |
|  |  | V181P | 2.2 | 50.8 ± 0.1 |
|  |  | V181R | 2.3 | 49.2 ± 0.01 |
|  |  | V181T | 2.4 | 55.1 ± 0.1 |

**Table S6.** Kinetic constants and deactivation constant of *Pc*LiP01 WT, V181A and E40S/V181A.

| **Parameters** | **WT** | **V181A** | **E40S/V181A** |
| --- | --- | --- | --- |
| *k*_d_ (min^-1^) | 3.67×10^-2^ ± 4.87×10^-3^ | 2.79×10^-3^ ± 2.74×10^-4^ | 1.25×10^-3^ ± 2.22×10^-4^ |
| *k*_cat_ at 25℃ (s^-1^) | 18.0 ± 0.15 | 19.1 ± 0.35 | 24.2 ± 0.28 |
| *K*_M_ at 25℃ (μM) | 80.3 ± 3.07 | 103 ± 8.01 | 126 ± 5.81 |
| *k*_cat_ at 60℃ (s^-1^) | 15.7 ± 3.42 | 69.7 ± 0.805 | 89.3 ± 1.64 |
| *K*_M_ at 60℃ (μM) | 1740 ± 713 | 346 ± 11.0 | 479 ± 22.3 |

**Table S7.** The average distance and interactions between the residue 181 and the heme propionate A from MD simulations of *Pc*LiP01 WT and V181X within the time range of 150–250 ns.

| **Variant** | **Backbone** | | **Sidechain** | | | | |
| --- | --- | --- | --- | --- | --- | --- | --- |
|  | **Carbon hydrogen bond** | | **Hydrogen bond** | | **Carbon hydrogen bond** | | **Salt bridge** |
|  | **Distance (Å)** | **Interactions** | **Distance (Å)** | **Interactions** | **Distance (Å)** | **Interactions** | **Interactions** |
| WT | 5.995 | 0.000 |  |  |  |  |  |
| V181A | 3.515 | 0.886 |  |  |  |  |  |
| V181E | 5.721 | 0.000 | 2.716 | 0.981 |  |  |  |
| V181F | 3.534 | 0.418 |  |  |  |  |  |
| V181H | 6.511 | 0.000 | 4.266 | 0.134 | 4.211 | 0.378 |  |
| V181I | 3.609 | 0.294 |  |  |  |  |  |
| V181L | 6.091 | 0.000 |  |  |  |  |  |
| V181M | 4.134 | 0.251 |  |  |  |  |  |
| V181Q | 3.585 | 0.821 | 5.669 | 0.000 |  |  |  |
| V181S | 3.364 | 0.228 | 2.644 | 1.000 | 3.388 | 0.000 |  |
| V181W | 3.530 | 0.864 | 4.932 | 0.000 | 3.709 | 0.679 |  |
| V181Y | 3.744 | 0.478 | 6.619 | 0.000 |  |  |  |

**Table S8.** Experimentally observed *T*_m_, predicted *T*_m_ by HemeLock Index, their difference and RZ for the WT and V181X variants of *Pc*LiP05 and *Pc*LiP09.

| **Enzyme** | **Variant** | **RZ** | ***T*_m_ (℃)** | | |
| --- | --- | --- | --- | --- | --- |
|  |  |  | **Observed** | **Predicted** | **Difference** |
| *Pc*LiP05 | WT | 3.4 | 53.7 ± 0.1 | 52.9 | -0.7 |
|  | V181A | 3.3 | 58.1 ± 0.1 | 58.8 | 0.7 |
|  | V181S | 2.7 | 55.9 ± 0.1 | 60.4 | 4.5 |
|  | V181T | 3.0 | 52.6 ± 0.3 | 56.3 | 3.7 |
|  | V181E | 2.8 | 50.1 ± 0.1 | 53.1 | 2.9 |
|  | V181H^*^ | 1.8 | 42.4 ± 0.1 | 53.8 | 11.4 |
| *Pc*LiP09 | WT | 2.7 | 54.6 ± 0.04 | 55.2 | 0.7 |
|  | V181A | 3.2 | 60.9 ± 0.1 | 62.4 | 1.5 |
|  | V181S | 3.6 | 58.2 ± 0.1 | 60.9 | 2.7 |
|  | V181Q | 3.2 | 55.5 ± 0.1 | 54.3 | -1.2 |
|  | V181T | 2.8 | 53.2 ± 0.2 | 59.7 | 6.6 |
|  | V181E | 2.4 | 52.9 ± 0.1 | 52.6 | 6.3 |
|  | V181Y | 1.7 | 48.5 ± 0.2 | 55.2 | 0.7 |
|  | V181H^*^ | 2.7 | 46.3 ± 0.2 | 62.4 | 1.5 |
|  | V181R | 1.2 | 44.9 ± 0.1 | 55.0 | 10.1 |

* Variants without activity.


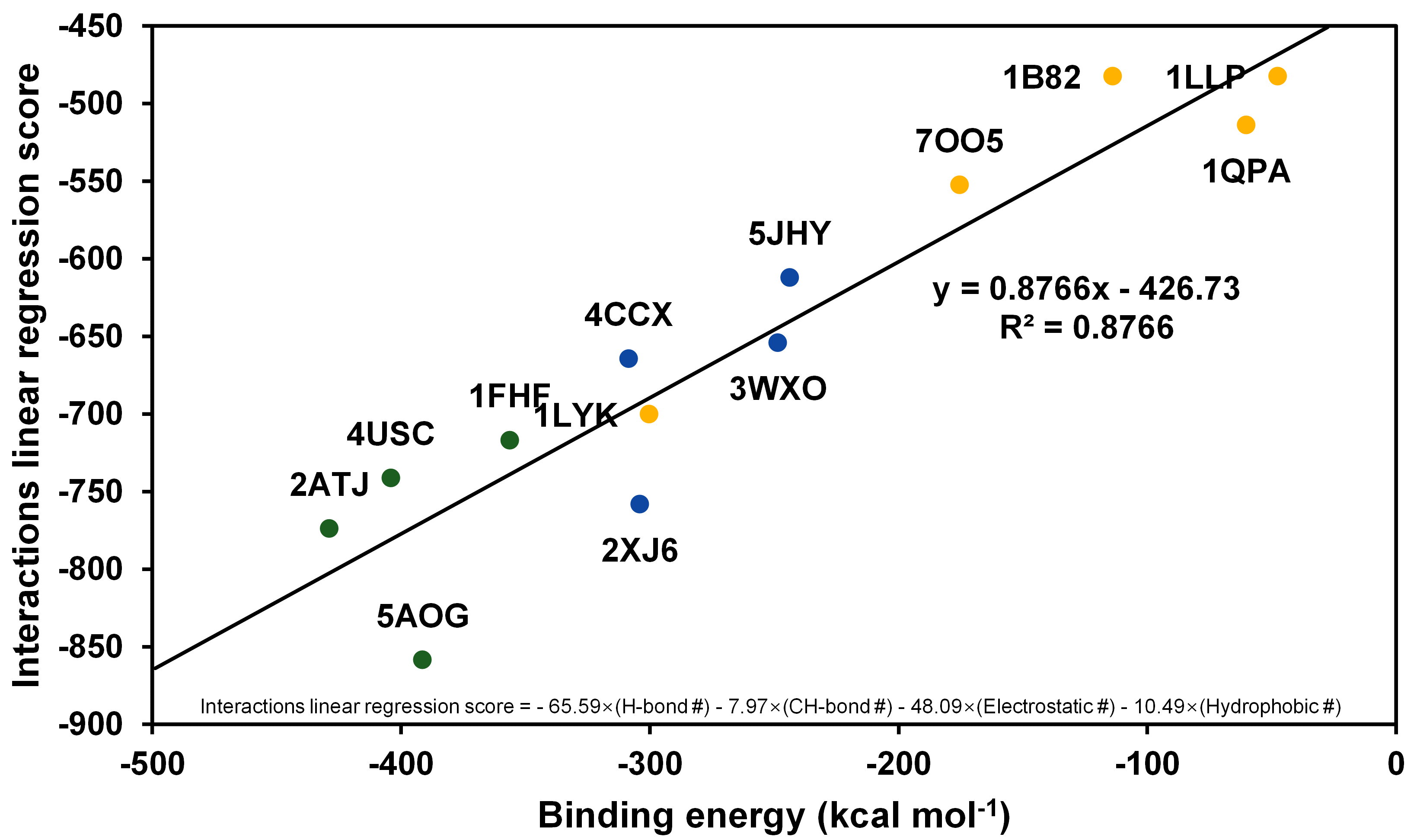


**Fig. S1.** The linear relationship between the heme-protein binding energy and the linear regression score of heme-protein non-bond interactions of heme enzymes.


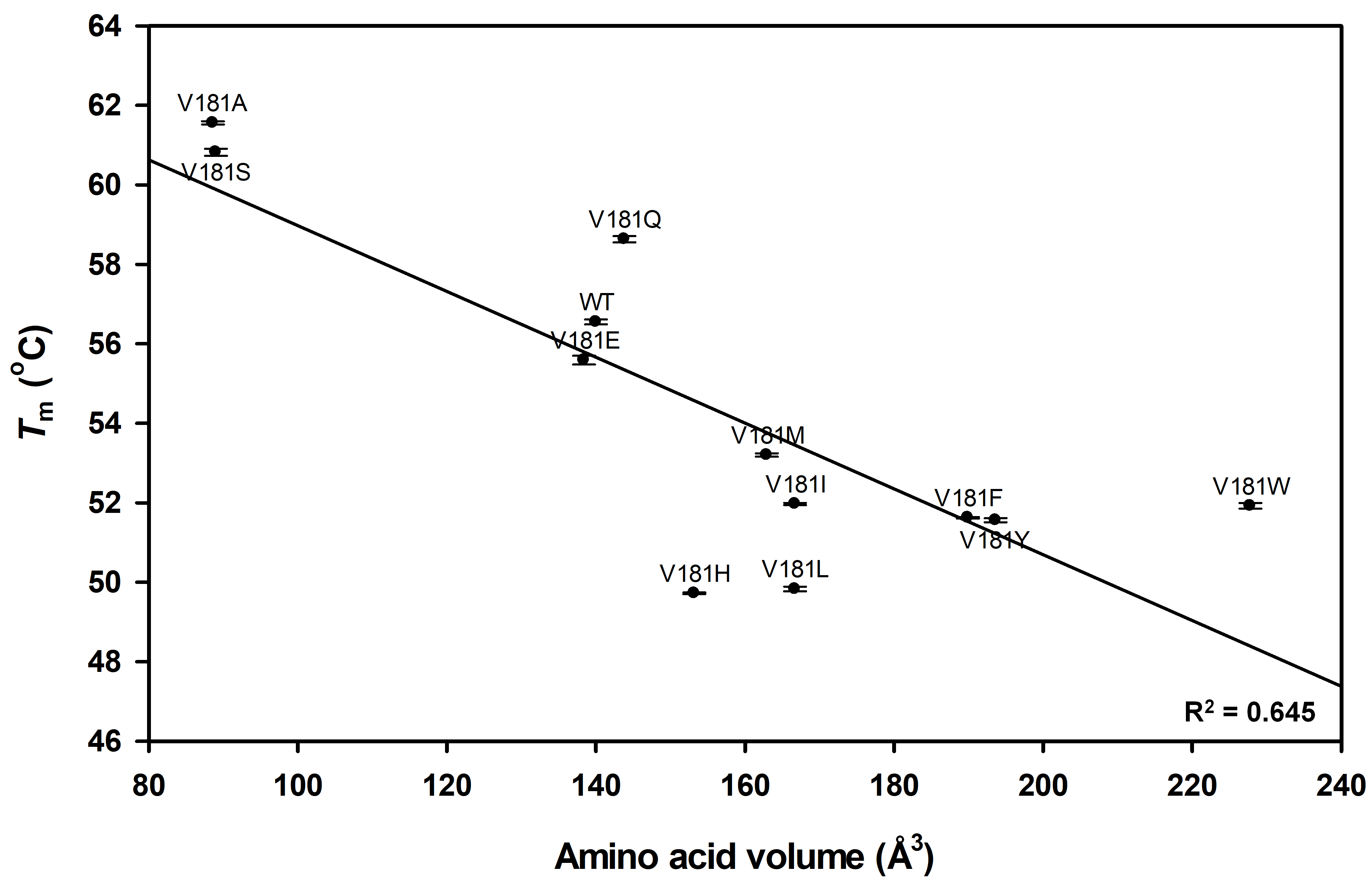


**Fig. S2.** The linear relationship between the volume of amino acid at residue 181 and *T*_m_ of *Pc*LiP01 V181X variants.


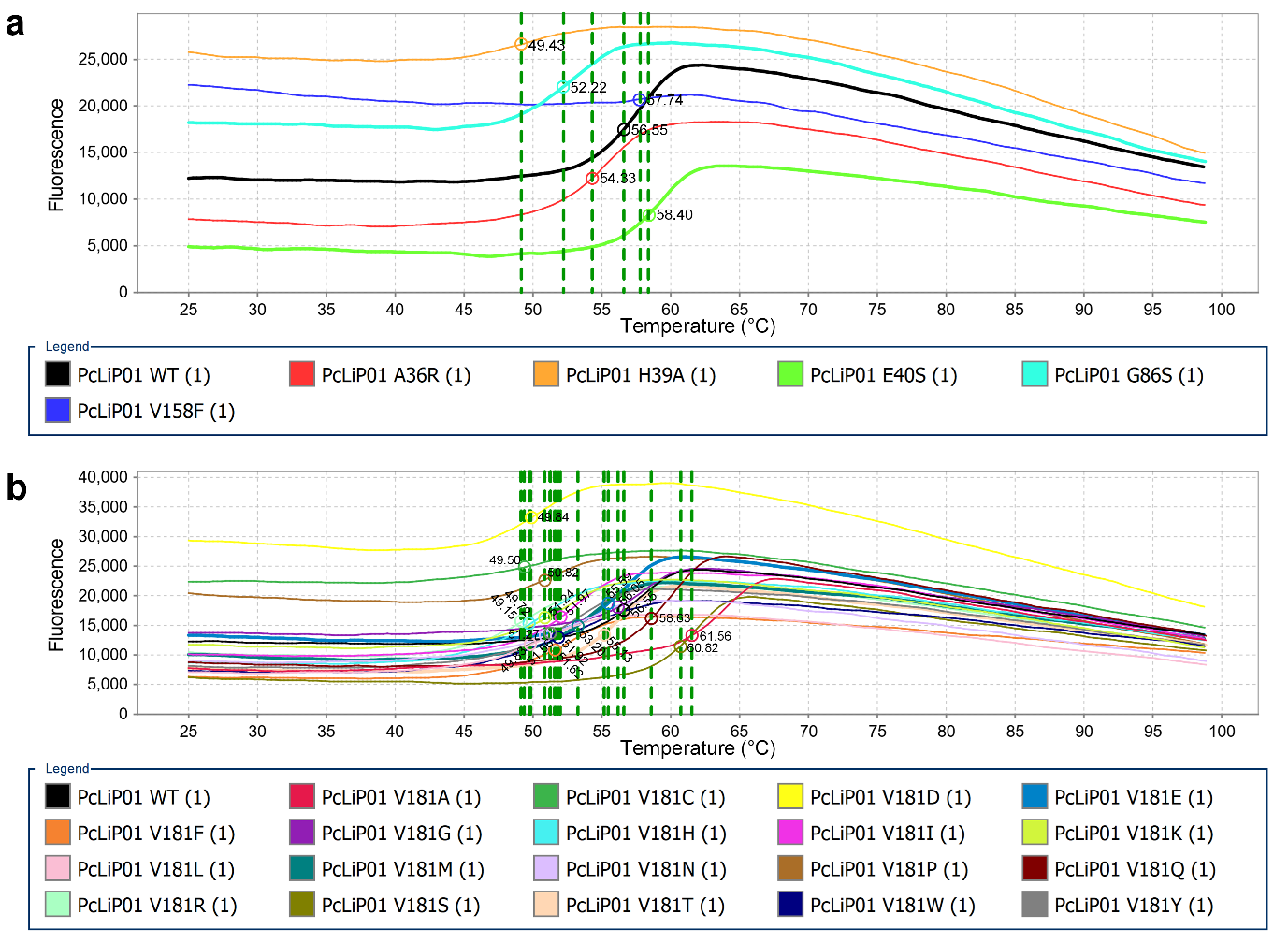


**Fig. S3.** Melting curves of *Pc*LiP01 wild type and homology mutants (a) or V181X saturation mutants (b). Green dashes indicate the Boltzmann *T*_m_ of each variants.


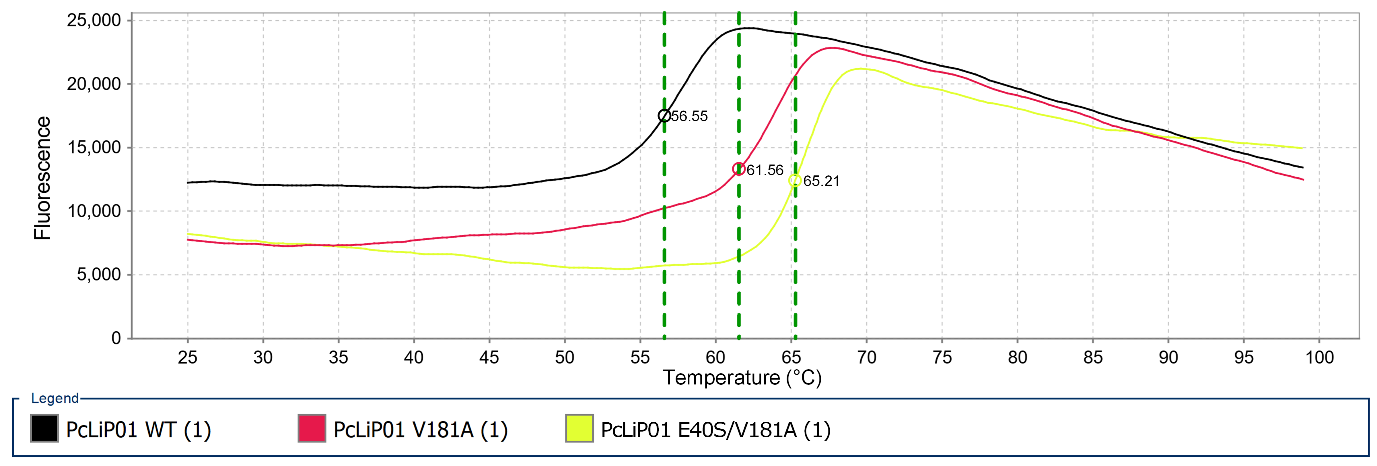


**Fig. S4.** Melting curves of *Pc*LiP01 WT, V181A and E40S/V181A. Green dashes indicate the Boltzmann *T*_m_ of each variants.


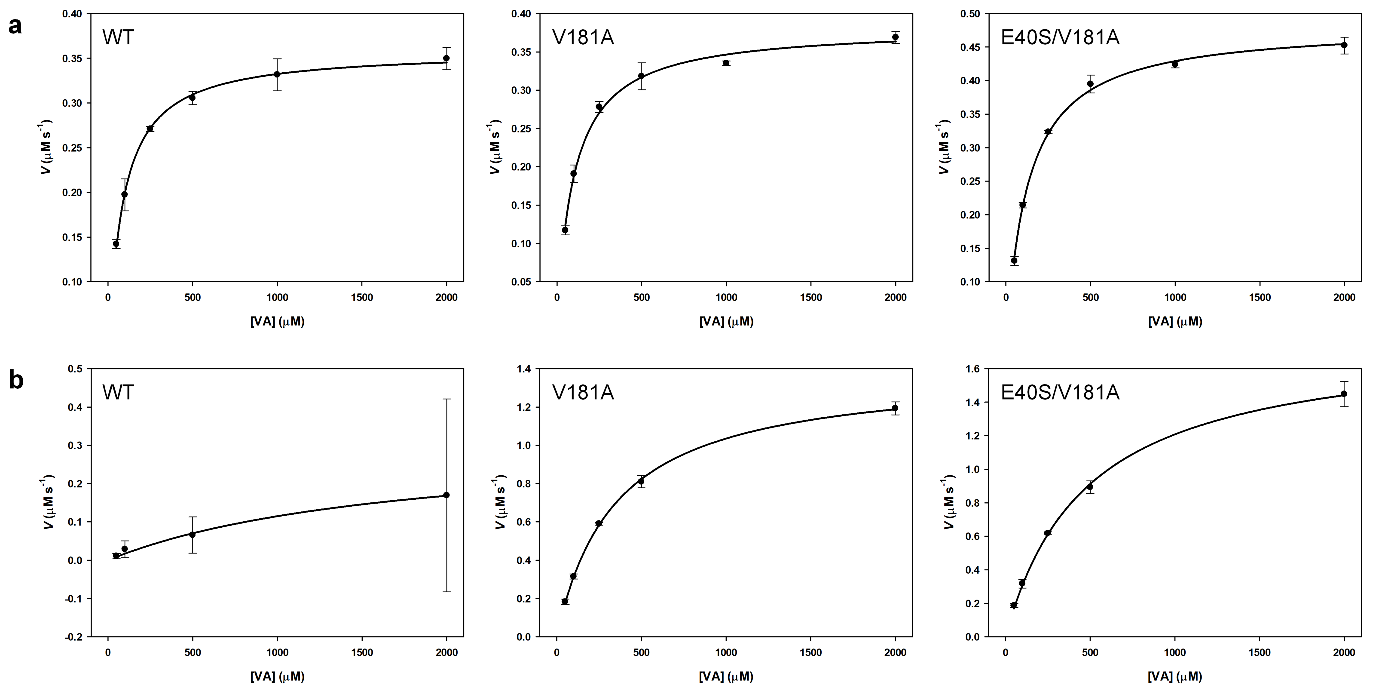


**Fig. S5.** Saturation curves of *Pc*LiP01 WT, V181A and E40S/V181A measured at 25℃ (a) or 60℃ (b).

**
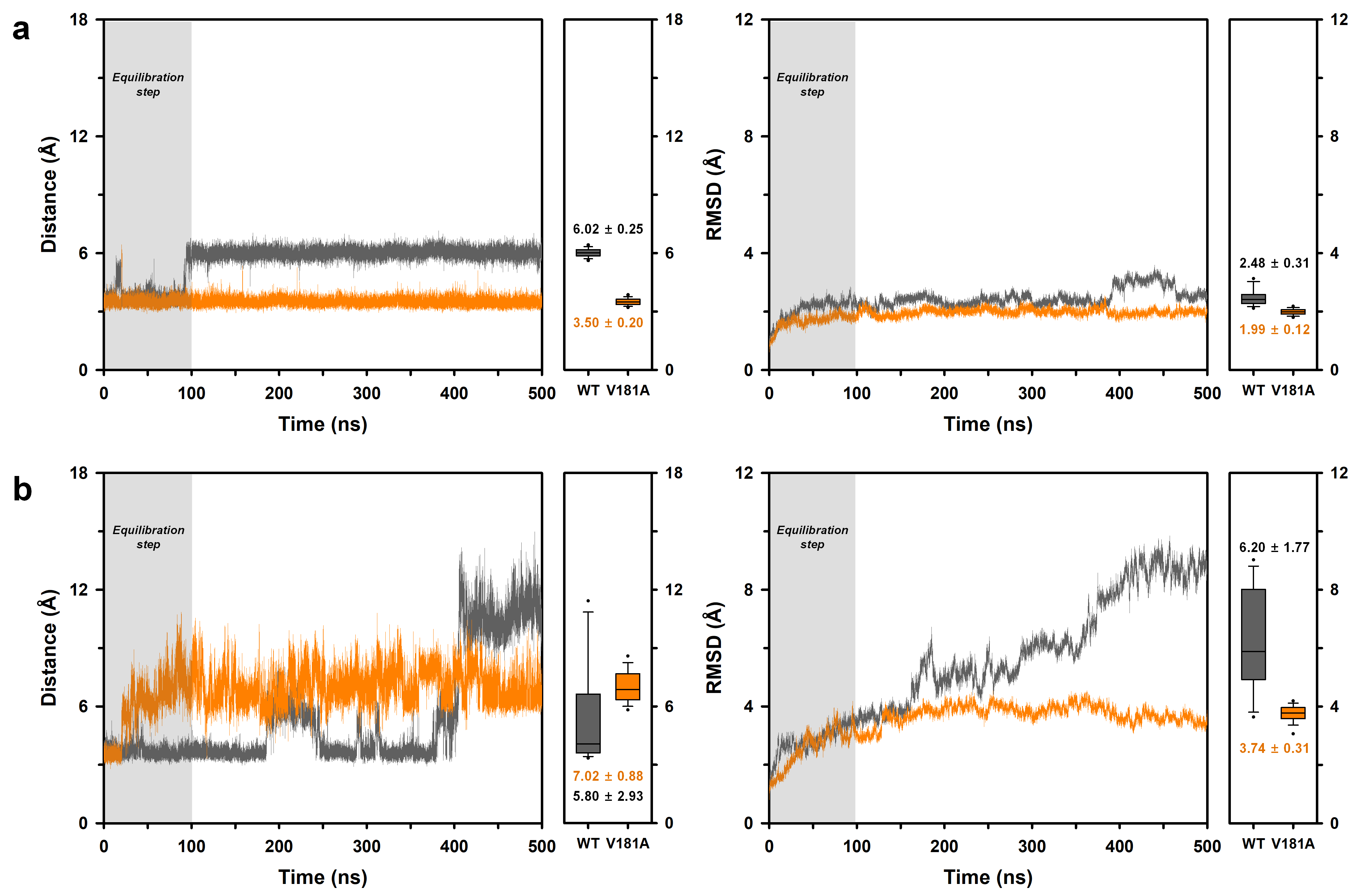
**

**Fig. S6.** MD simulation data at 25℃ and 80℃ for *Pc*LiP01 WT (gray) and V181A (orange) over time. Data until 100 ns were regarded as equilibration steps. Box plots represent the distribution of distance or RMSD after equilibration steps by plotting the median, 10th, 25th, 75th, and 90th percentiles as vertical boxes with error bars. Dots represent 5th and 95th percentiles. (a) Distance between the oxygen of heme propionate A and the Cα atom of the residue 181, and overall backbone RMSD at 25℃. (b) Distance between the oxygen of heme propionate A and the Cα atom of the residue 181, and overall backbone RMSD at 80℃.


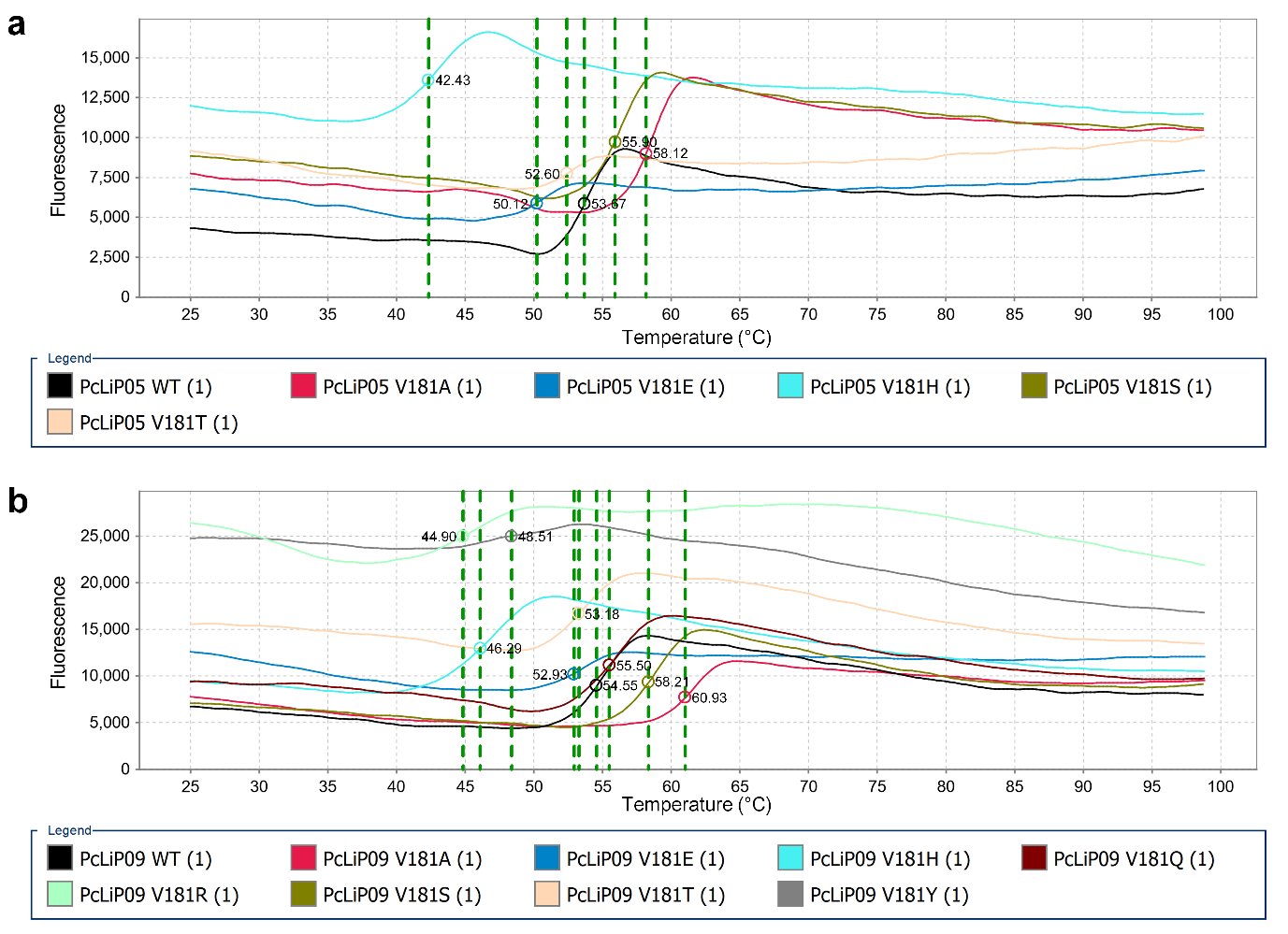


**Fig. S7.** Melting curves of WT and V181X variants of *Pc*LiP05 (a) and *Pc*LiP09 (b). Green dashes indicate the Boltzmann *T*_m_ of each variants.

**
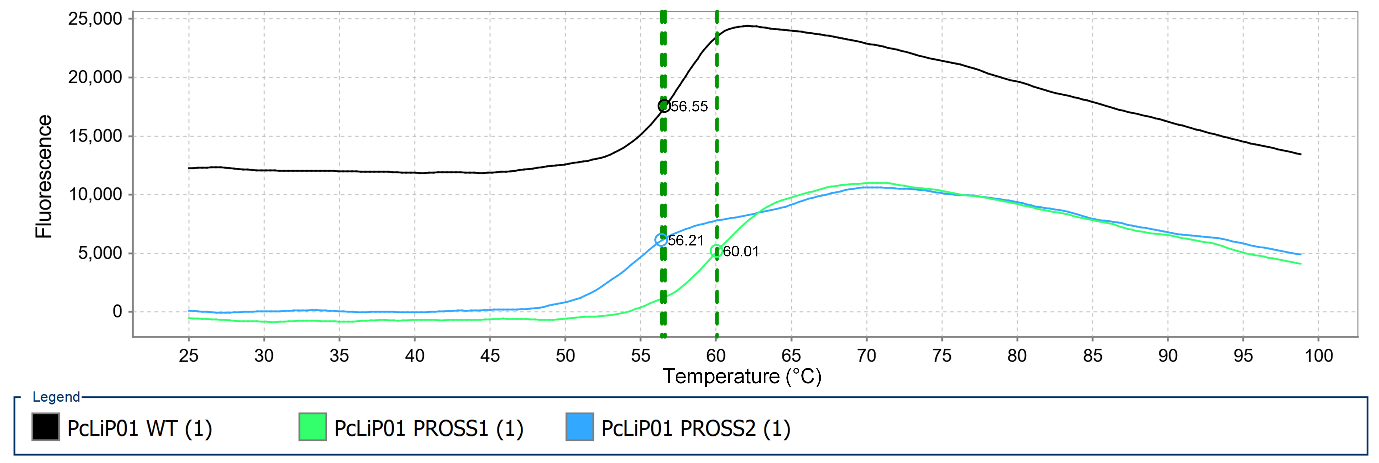
**

**Fig. S8.** Melting curves of *Pc*LiP01 WT and PROSS variants. Green dashes indicate the Boltzmann *T*_m_ of each variants.

**
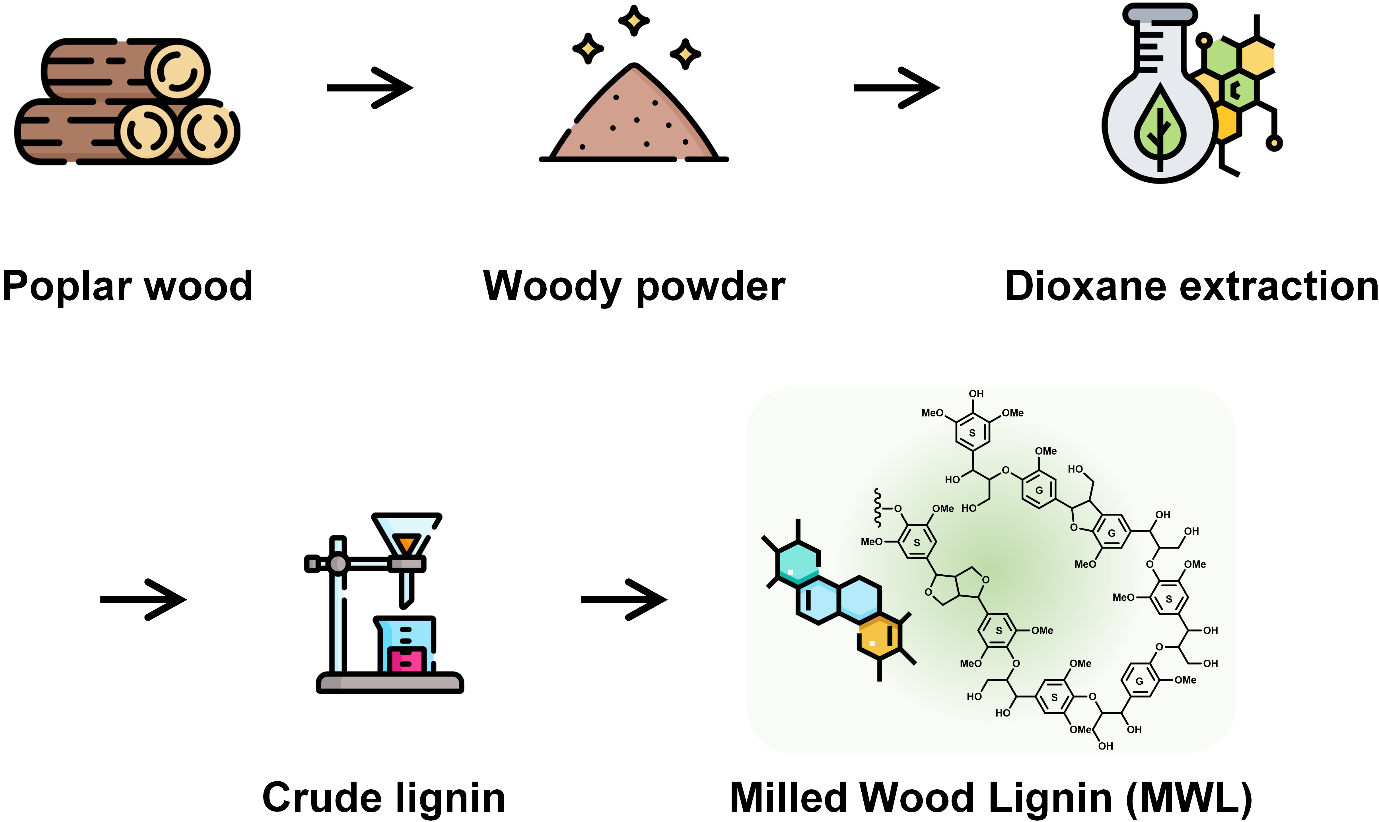
**

**Fig. S9.** Preparation of milled wood lignin.


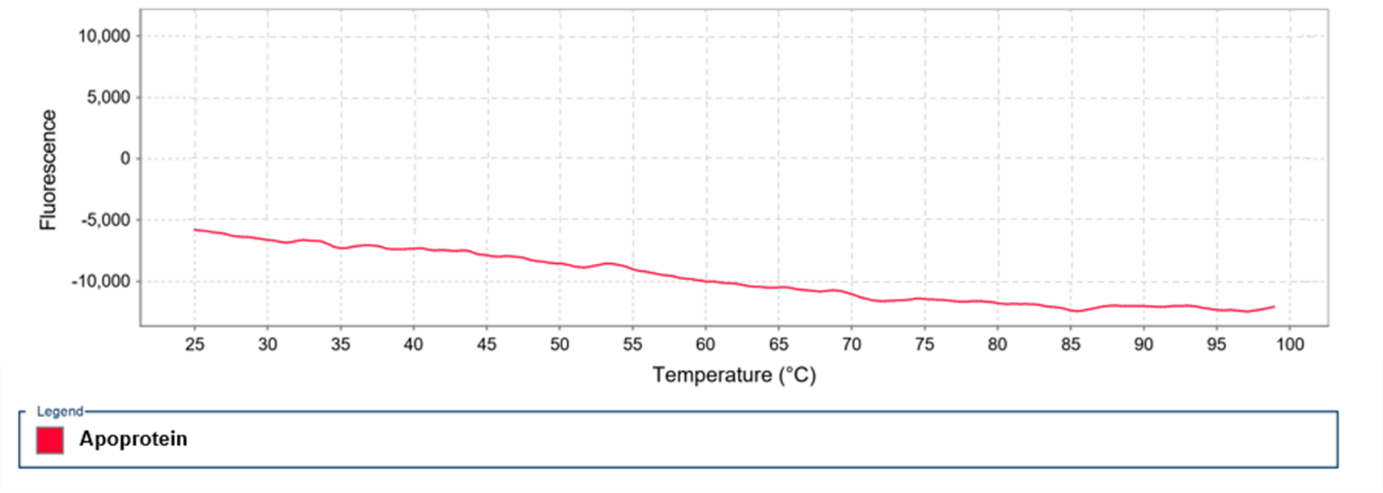


**Fig. S10.** Melting curve of apo-*Pc*LiP01 WT.

**References**

[1] J.L. Markley, A. Bax, Y. Arata, C.W. Hilbers, R. Kaptein, B.D. Sykes, P.E. Wright, K. Wüthrich, Recommendations for the presentation of NMR structures of proteins and nucleic acids (IUPAC recommendations 1998), Pure Appl. Chem. 70 (1998) 117–142. https://doi.org/10.1351/pac199870010117.

[2] D.K. Jones, D.A. Dalton, F.I. Rosell, E.L. Raven, Class I heme peroxidases: characterization of soybean ascorbate peroxidase, Arch. Biochem. Biophys. 360 (1998) 173–178. https://doi.org/10.1006/abbi.1998.0941.

[3] B. Gasselhuber, M.M.H. Graf, C. Jakopitsch, M. Zamocky, A. Nicolussi, P.G. Furtmüller, C. Oostenbrink, X. Carpena, C. Obinger, Interaction with the redox cofactor MYW and functional role of a mobile arginine in eukaryotic catalase-peroxidase, Biochemistry 55 (2016) 3528–3541. https://doi.org/10.1021/acs.biochem.6b00436.

[4] S. Kamachi, K. Hirabayashi, M. Tamoi, S. Shigeoka, T. Tada, K. Wada, Crystal structure of the catalase–peroxidase KatG W78F mutant from *Synechococcus elongatus* PCC7942 in complex with the antitubercular pro-drug isoniazid, FEBS Lett. 589 (2014) 131–137. https://doi.org/10.1016/j.febslet.2014.11.037.

[5] Y.G.J. Sterckx, A.N. Volkov, Cofactor-dependent structural and binding properties of yeast cytochrome *c* peroxidase, Biochemistry 53 (2014) 4526–4536. https://doi.org/10.1021/bi500603w.

[6] J.P. McEldoon, J.S. Dordick, Unusual thermal stability of soybean peroxidase, Biotechnol. Progr. 12 (1996) 555–558. https://doi.org/10.1021/bp960010x.

[7] M.I. Sánchez-Ruiz, I. Ayuso-Fernández, J. Rencoret, A.M. González-Ramírez, D. Linde, I. Davó-Siguero, A. Romero, A. Gutiérrez, A.T. Martínez, F.J. Ruiz-Dueñas, Agaricales mushroom lignin peroxidase: from structure–function to degradative capabilities, Antioxidants 10 (2021) 1446. https://doi.org/10.3390/antiox10091446.

[8] H. Gye, H. Baek, S. Han, H. Kwon, V.T.T. Nguyen, L.T.M. Pham, S. Kang, Y.H. Nho, D.W. Lee, Y.H. Kim, Recombinant lignin peroxidase with superior thermal stability and melanin decolorization efficiency in a typical human skin-mimicking environment, Biomacromolecules 24 (2023) 2633–2642. https://doi.org/10.1021/acs.biomac.3c00123.

[9] L.S. Zamorano, S.B. Vilarmau, J.B. Arellano, G.G. Zhadan, N.H. Cuadrado, S.A. Bursakov, M.G. Roig, V.L. Shnyrov, Thermal stability of peroxidase from *Chamaerops excelsa* palm tree at pH 3, Int. J. Biol. Macromol. 44 (2009) 326–332. https://doi.org/10.1016/j.ijbiomac.2009.01.004.

[10] M.H. Dicko, H. Gruppen, R. Hilhorst, A.G.J. Voragen, W.J.H. van Berkel, Biochemical characterization of the major sorghum grain peroxidase, FEBS J. 273 (2006) 2293–2307. https://doi.org/10.1111/j.1742-4658.2006.05243.x.

[11] J.K.A. Kamal, D.V. Behere, Thermal and conformational stability of seed coat soybean peroxidase, Biochemistry 41 (2002) 9034–9042. https://doi.org/10.1021/bi025621e.
